# Design and Synthesis of a Chiral Halogen-Bond Donor with a Sp^3^-Hybridized Carbon–Iodine Moiety in a Chiral Fluorobissulfonyl Scaffold

**DOI:** 10.3390/molecules25194539

**Published:** 2020-10-03

**Authors:** Hiroto Uno, Kohei Matsuzaki, Motoo Shiro, Norio Shibata

**Affiliations:** 1 Department of Nanopharmaceutical Sciences, Nagoya, Institute of Technology, Gokiso, Showa-ku, Nagoya 466-8555, Japan; h.uno.844@stn.nitech.ac.jp (H.U.); kohei-matsuzaki@oat-agrio.co.jp (K.M.); 2 Rigaku Corporation, 3-9-12, Matsubara-cho, Akishima-shi, Tokyo 196-8666, Japan; msshiro@nyc.odn.ne.jp; 3 Institute of Advanced Fluorine-Containing Materials, Zhejiang Normal University, 688 Yingbin Avenue, Jinhua 321004, China

**Keywords:** halogen-bond, chiral halogen-bond donor, Lewis acid, organo-catalyst

## Abstract

The first example of a chiral halogen-bond donor with a sp^3^-hybridized carbon–iodine moiety in a fluorobissulfonyl scaffold is described. The binaphthyl backbone was designed as a chiral source and the chiral halogen-bond donor (*R*)-**1** was synthesized from (*R*)-1,1′-binaphthol in 11 steps. An NMR titration experiment demonstrated that (*R*)-**1** worked as a halogen-bond donor. The Mukaiyama aldol reaction and quinoline reduction were examined using (*R*)-**1** as a catalyst to evaluate the asymmetric induction.

## 1. Introduction

**Figure 1:**
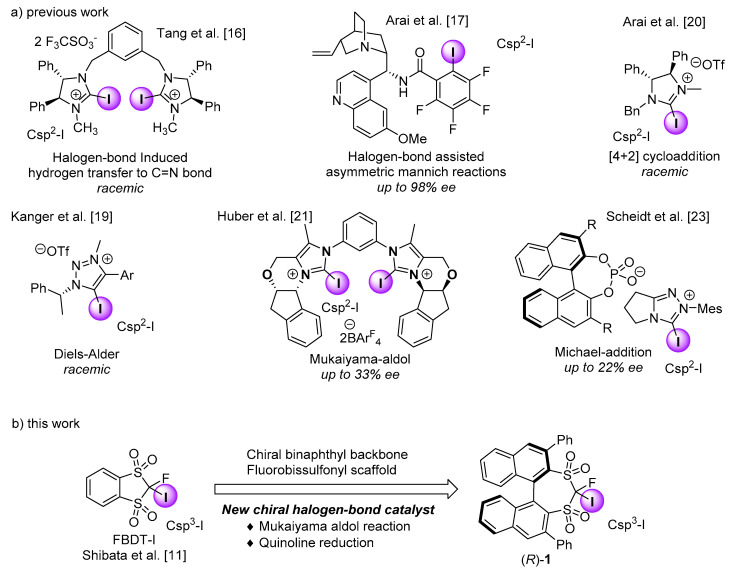
The use of chiral halogen-bond donors as organo-catalysts: (**a**) previous work, (**b**) this work.

Halogen-bonds (XBs) are one type of non-covalent interactions between an electrophilic region associated with a halogen atom and a Lewis base [1,2]. The strength of halogen bonding interactions is similar to those of hydrogen bonds (HBs), encouraging us to investigate XBs as a new driving force for organic reactions. Recently, reactions using halogen-bond donors as catalysts have been reported [3,4], for example, reduction reaction [5], halide abstraction [6,7], Diels-Alder [8,9], and Michael addition [10]. Our group also reported that FBDT-I (2-fluoro-1,3-benzodithiole-1,1,3,3-tetraoxy-2-iodide), a halogen-bond donor with a sp^3^-hybridized carbon–iodine moiety, catalyzed the Mukaiyama aldol reaction and reduction of quinolines [11]. Increasing attention has been paid to achiral reactions as well as the application of XBs in asymmetric synthesis [12,13,14,15]. The strong directionality of XBs can be considered more advantageous than HBs, which have been extensively used in asymmetric catalysis. This property makes it possible to form rigid asymmetric environments around substrates activated by XBs. Thus, chiral XB donors have good potential as asymmetric catalysts. In 2004, Tang et al. synthesized chiral cationic imidazolium XB donors, which catalyzed hydrogen transfer reactions to C=N bonds (but no asymmetric induction) [16]. Arai et al. accomplished halogen-bond-assisted asymmetric Mannich reactions using chiral XB catalysts derived from cinchona alkaloids (Figure 1a) [17,18]. Although four more chiral XB catalysts were reported afterwards [19,20,21,22,23], all the structures of chiral XB catalysts are limited to those containing a sp^2^-hybridized carbon–iodine (C_sp2_–I) moiety. Besides, the current chiral XB catalysts are based on very few core structures, resulting in less developed asymmetric reactions, and thus a need for novel motifs. In this context, we were interested in the novel chiral XB catalysts containing a sp^3^-hybridized carbon–iodine (C_sp3_–I) moiety. A C_sp3_–I moiety in chiral XB catalysts would construct an asymmetric carbon center directly attached to iodine. The sp^3^-hybridized carbon–iodine chiral center should be advantageous for creating complex three-dimensional environments around the iodine, which play a critical role for XB. However, the chiral XB catalysts containing a C_sp3_–I moiety have yet to be reported. Herein, we report a novel chiral halogen-bond donor with a C_sp3_–I moiety in a chiral fluorobissulfonyl scaffold, and its attempt to use the Mukaiyama aldol reaction and quinoline reduction for evaluation of its catalytic activity (Figure 1b).

## 2. Results and Discussion

**Figure 2:**
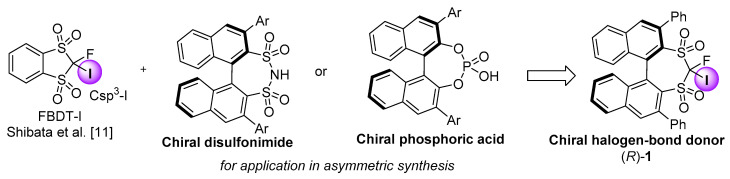
Design of a chiral halogen-bond donor (*R*)-**1** with a sp^3^-hybridized carbon–iodine moiety.

We recently reported the development of new halogen-bond donors with fluorobissulfonyl scaffolds and their application as a Lewis-acid catalyst in the Mukaiyama aldol reaction [11]. Our designed halogen-bond donor, FBDT-I, can be synthesized easily, which makes it possible to design its chiral derivatives. Thus, as an extension of our halogen-bond chemistry, we wanted to develop a new chiral halogen-bond donor. In our previous report [11], we disclosed that FBDT-I catalyzed the reduction reaction of quinolines with the Hantzsch ester. An asymmetric path to achieving this type of reduction using chiral phosphoric acid catalysts, which have binaphthyl moieties, was reported by Rueping et al. [24]. List et al. also reported the enantioselective reduction of imines using chiral disulfonimide as a Brønsted acid catalyst [25]. These facts encouraged us to assume that a fuluorobissulfonyl halogen-bond donor, which has a chiral binaphthyl moiety, could also work as an asymmetric catalyst. Thus, we designed a fluorobissulfonyl halogen-bond donor **1** with a chiral binaphthyl backbone (Figure 2).

**Scheme 1:**
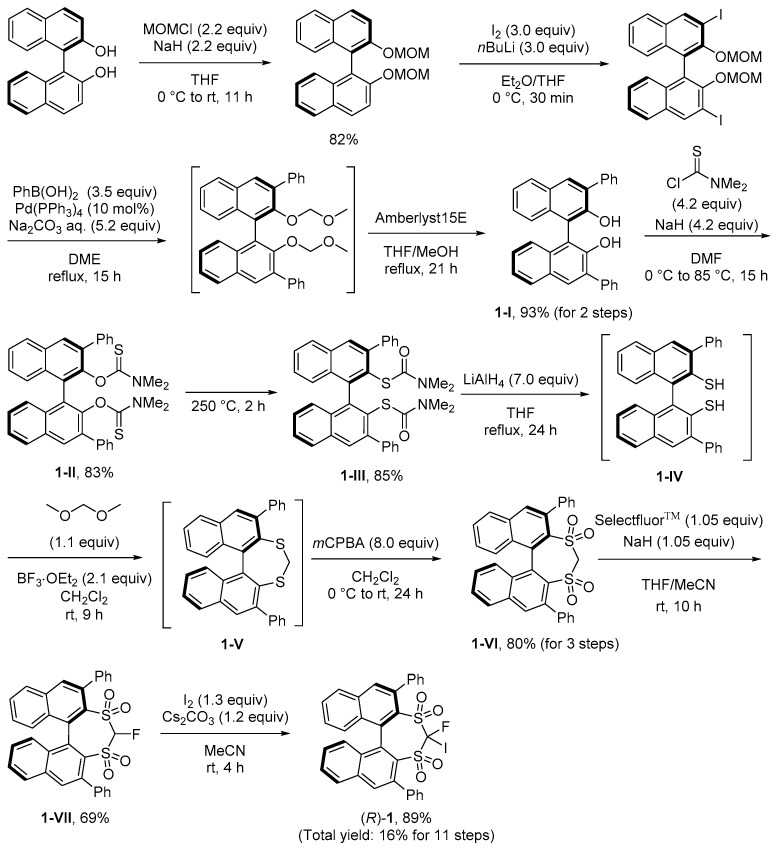
Synthesis of chiral halogen-bond donor (*R*)-**1**.

The synthetic route of the chiral binaphthyl halogen-bond donor (*R*)-**1** is shown in Scheme 1. 3,3′-Phenyl-substituted binaphthol **1-I** was synthesized from (*R*)-1,1′-binaphthol according to a reported procedure [26]. Then, carbamoylation of the hydroxy group gave **1-II**, and a Newman-Kwart rearrangement afforded thiocarbamate **1-III** in 85% yield. Next, reduction using LiAlH_4_ gave dithiol **1-IV**, alkylation of thiol with dimethoxymethane afforded cyclic sulfide **1-V**, and bissulfonylmethane **1-VI** was synthesized by *m*CPBA (*m*-chloroperoxybenzoic acid) oxidation in 80% yield within three steps. The fluorination of **1-VI** with Selectfluor^TM^ and iodination of **1-VII** under basic conditions gave product (*R*)-**1** in good yield (69%, 89%) (see Supplementary Materials). Although the total yield for the preparation of (*R*)-**1** was good (16% for 11 steps), the synthesis of this catalyst having a C_sp3_–I moiety was rather complicated for the preparation of other chiral XB catalysts with a C_sp2_–I moiety [17,18,19,20,21,22,23].

**Figure 3:**
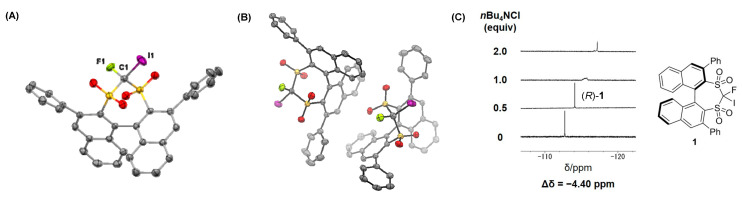
(**A**) X-ray structure of (*R*)-**1** with ellipsoid at 50% probability (CCDC 2031026). The hydrogen atoms and solvent were omitted for clarity. Selected bond distances (Å): C1–I1, 2.130 (4), C1–F1, 1.370 (5). (**B**) X-ray packing structure of (*R*)-**1** with ellipsoid at 50% probability. The hydrogen atoms and solvent were omitted for clarity. (**C**) ^19^F NMR titration of (*R*)-**1** with *n*Bu_4_NCl in CDCl_3_.

The structure of (*R*)-**1** was determined by X-ray analysis of a single crystal of (*R*)-**1** from the recrystallization of (*R*)-**1** in CH_2_Cl_2_ (1,2-dichloroethane)/hexane (Figure 3A, unit cell parameters: a = 10.6720(2), b = 10.9407(2), c = 13.7402(3), α = 90, β = 106.390(8), γ = 90). The length of carbon–iodine was 2.130 Å, corresponding to the general C_sp3_–I bond length (2.13 Å). For future design, we assumed that the longer substituted groups at 3,3′-position of binaphthyl might be better for asymmetric induction, because the XB-activated substrates could not be positioned near the chiral environment of the catalyst due to the long distance of C_sp3_–I. In a previous report [11], we revealed that intermolecular halogen bonding between the iodine atom and the oxygen atom of the sulfonyl group was formed in FBDT-I. However, in this chiral (*R*)-**1**, intermolecular halogen bonding was not observed (Figure 3B). We believe that it was due to the steric repulsion of (*R*)-**1**. Next, we tried to observe the halogen-bond in the solution state. Figure 3C shows the result of the NMR titration experiments on (*R*)-**1** with tetrabutylammonium chloride (*n*Bu_4_NCl) in chloroform-d (CDCl_3_) (Figure 3C). After titration, the ^19^F NMR signals were upshifted (−4.40 ppm), corresponding with our previous report [11]. This result indicates that the iodine atom of (*R*)-**1** interacts with the chloride anion of *n*Bu_4_NCl. Thus, it was demonstrated that (*R*)-**1** works as a halogen-bond donor.

**Scheme 2:**
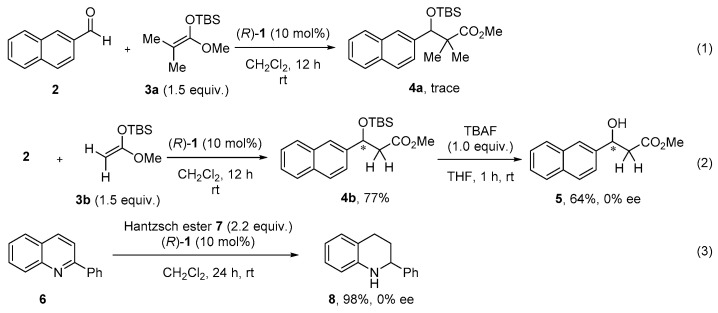
Mukaiyama aldol reaction (Equations (1) and (2)) and reduction of quinoline (Equation (3)) with (*R*)-**1**.

Encouraged by this potential of (*R*)-**1**, we examined (*R*)-**1** as a catalyst in the Mukaiyama aldol reaction with dimethyl-substituted silyl ketene acetal **3a**, as in our previous report [11]. Surprisingly, only trace amounts of product **4a** were formed in the reaction (Scheme 2(1)). We assumed that the steric repulsion between (*R*)-**1** and **3a** inhibited the nucleophilic attack on aldehyde **2**. Thus, we attempted the reaction with less steric non-substituted silyl ketene acetal **3b**, and product **4b** was successfully obtained in 77% yield. However, HPLC analysis of desilylated **5** revealed that stereoselectivity was not observed (Scheme 2(2)). Next, reduction of quinoline **6** with a catalytic amount of (*R*)-**1** was examined, and product **8** was obtained in 98% yield, but the product was racemic (Scheme 2(3)).

**Scheme 3:**
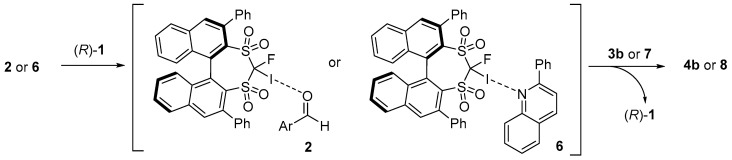
A plausible reaction mechanism for the transformation from **2** or **6** into **4b** or **8** using (*R*)-**1**.

A plausible reaction mechanism is shown in Scheme 3. In the initial step, the halogen-bond donor (*R*)-**1** works as a Lewis acid to activate the carbonyl group of aldehyde **2** or the nitrogen atom of quinolone **6**. The nucleophile **3b** or hydride can then attack substrates **2**/**6** to form the desired products **4b**/**8** (Scheme 3). The lack of asymmetric induction suggests that the monodentate structure of **1** is not suitable for asymmetric catalysis.

## 3. Materials and Methods

### 3.1. General Information

All reactions were performed in oven-dried glassware under a positive pressure of nitrogen. Solvents were transferred via syringe and were introduced into the reaction vessels through a rubber septum. Chemicals were purchased and used without further purification unless otherwise noted. All of the reactions were monitored by thin-layer chromatography (TLC) carried out on a 0.25 mm Merck silica gel (60-F254) (Kenilworth, NJ, USA). TLC plates were visualized with UV light and 7% phosphomolybdic acid or KMnO_4_ in water/heat. Column chromatography was carried out on a column packed with silica gel (60 N spherical neutral size 50–60 μm) supplied by Kanto Chemical Co., Inc. (Tokyo, Japan). The ^1^H-NMR (300 MHz), ^19^F-NMR (282 MHz), and ^13^C-NMR (126 MHz) spectra for each solution in CDCl_3_ were recorded on Varian Mercury 300 (Agilent Technologies, Palo Alto, CA, USA) and Avance 500 (Bruker, Billerica, MA, USA) NMR spectrometers. Chemical shifts (δ) are expressed in ppm downfield from TMS (δ = 0.00) or C_6_F_6_ (δ = −162.2 (CDCl_3_)) as an internal standard. Mass spectra were recorded on a SHIMADZU GCMS-QP5050A (EI-MS) and SHIMAZU LCMS-2020 (ESI-MS) (Shimadzu Corporation, Kyoto, Japan). Melting points were recorded on Buchi M-565. Infrared spectra were recorded on a JASCO FT/IR-4100 spectrometer. Chemicals were purchased and used without further purification unless otherwise noted. CH_2_Cl_2_ was dried and distilled before use. X-ray measurements were carried out on a Rigaku R-AXIS RAPID or Rigaku Mercury70 diffractometer with graphite monochromated Mo Kα radiation at −100 °C. The crystal structure was solved by the direct method (SIR2004) and refined by the full-matric least-square technique (SHELXL97). The non-hydrogen atoms were refined anisotropically. Hydrogen atoms were refined by the riding model using the appropriate HFIX command in SHELXL97. All calculations were performed with the CrystalStructure software package.

### 3.2. Synthesis of the Chiral Halogen-Bond Donor (R)-***1***

*(R)-O,O’-(3,3′-Diphenyl-[1,1′-binaphthalene]-2,2′-diyl) bis(dimethylcarbamothioate)* (**1-II**)

(*R*)-3,3′-diphenyl-BINOL (**1-I**) was prepared according to published literature [26]. 

**1-II** was prepared according to published literature [27]. 

To a solution of (*R*)-3,3′-diphenyl-BINOL (2.71 g, 6.18 mmol, 1.0 equiv.) in *N*,*N*-dimethylformamide (DMF) (20 mL) cooled by ice/water bath, dropwise a suspension of sodium hydride (NaH) was added (60% oil suspension, 1.04 g, 26.0 mmol, 4.2 equiv.) in DMF (7.0 mL) and the mixture was stirred at 0 °C for 10 min. A solution of dimethylthiocarbamoyl chloride (2.80 g, 26.0 mmol, 4.2 equiv.) in DMF (7.0 mL) was added, and the mixture was allowed to warm to room temperature over 1 h and then stirred at 85 °C for 43 h. After cooling to room temperature, aqueous 3% KOH solution (56 mL) was added, and the resulting precipitate was filtered and washed with H_2_O. The precipitate was dissolved into CH_2_Cl_2_, and the solution was washed with H_2_O and brine, dried over Na_2_SO_4_, and concentrated under reduced pressure. The crude product was purified by column chromatography on silica gel (CHCl_3_) to give product **1-II** (3.14 g, 83% yield) as a white solid.

Several peak sets were observed due to the rotamers of the title compound. All peaks are given below:

mp = 138.7–139.7 °C (CHCl_3_); MS (ESI, *m*/*z*) 623 [(M + Na)^+^]; HRMS (ESI) calcd. for C_38_H_32_N_2_NaO_2_S_2_ [(M + Na)^+^]: 635.1803 found 635.1815; ^1^H NMR (CDCl_3_, 500 MHz): δ = 2.04 (s, 6H rotamer), 2.68 (s, 6H rotamer), 2.73 (s, 6H rotamer), 2.82 (s, 6H rotamer), 2.87 (s, 6H rotamer), 3.03 (s, 6H rotamer), 7.21–8.07 (m, 20H); ^13^C NMR (CDCl_3_, 126 MHz): δ = 37.0, 37.9, 39.0, 42.2, 42.6, 125.0, 125.7, 125.8, 125.9, 126.05, 126.08, 126.2, 126.5, 127.0, 127.19, 127.25, 127.3, 127.6, 127.9, 127.99, 128.02, 128.1, 128.4, 128.5, 129.3, 129.46, 129.49, 129.7, 130.1, 130.4, 131.6 131.7, 132.5, 132.7, 133.4, 134.9, 135.4, 136.5, 138.33, 138.36, 139.0, 147.4, 147.5, 148.1, 184.7, 185.4, 185.7; IR (KBr) 699, 732, 756, 789, 892, 910, 1119, 1137, 1152, 1184, 1243, 1286, 1359, 1392, 1420, 1451, 1497, 1530, 2935, 3053 cm^−1^.

*(R)- S,S’-(3,3′-Diphenyl-[1,1′-binaphthalene]-2,2′-diyl) bis(dimethylcarbamothioate)* (**1-III**)

**1-III** was prepared according to published literature [27].

A test tube equipped with rubber septa and magnetic stir bar was charged with **1-II** (1.5 g, 2.45 mmol, 1.0 equiv.). The tube was degassed by vacuum evacuation and backfilled with argon. The tube was placed into a preheated (250 °C) sand bath and stirred at 250 °C for 2 h. After cooling to room temperature, CHCl_3_ was added, and the crude was purified by column chromatography on silica gel (CHCl_3_) to give the desired product **1-III** (1.27 g 85% yield) as a slightly yellow solid.

Several peak sets were observed due to the rotamers of the title compound. All peaks are given below:

mp = 243.6–244.3 °C (CHCl_3_); MS (ESI, *m*/*z*) 613 [(M + Na)^+^]; HRMS (ESI) calcd. for C_38_H_32_N_2_NaO_2_S_2_ [(M + Na)^+^]: 635.1803 found 635.1803; ^1^H NMR (CDCl_3_, 300 MHz): δ = 2.42 (s, 12H), 7.22–7.49 (m, 12H), 7.59–7.63 (m, 4H), 7.88 (d, *J* = 8.1 Hz, 2H), 7.96 (s, 2H); ^13^C NMR (CDCl_3_, 126 MHz): δ = 38.6, 126.1, 126.8, 127.1, 127.2, 127.5, 127.9, 128.1, 129.4, 130.2, 132.4, 133.5, 142.0, 144.1, 144.3, 165.5; IR (KBr) 527, 606, 653, 682, 700, 751, 783, 907, 1027, 1090, 1259, 1359, 1403, 1444, 1492, 1666, 2927, 3026, 3052 cm^−1^.

*(R)-2,6-Diphenyl-4H-dinaphtho [2,1-d:1′,2′-f][1,3]dithiepine 3,3,5,5-tetraoxide* (**1-VI**)

**1-VI** was prepared according to published literature [27].

Step 1:

To a solution of **1-III** (1.25 g, 2.04 mmol, 1.0 equiv.) in tetrahydrofuran (THF) (30 mL), portionwise lithium aluminum hydride (543 mg, 14.3 mmol, 7.0 equiv.) was added and the mixture was stirred at reflux (80 °C) for 24 h. After cooling to room temperature, the reaction was quenched by aqueous 10% HCl solution (60 mL) and extracted with CH_2_Cl_2_. The combined organic layer was dried over Na_2_SO_4_ and concentrated under reduced pressure. The crude product **1-IV** (836 mg, 87% yield, slightly yellow solid) was used in the next reaction without further purification.

Step 2:

To a solution of **1-IV** (836 mg, 1.78 mmol, 1.0 equiv.) and dimethoxymethane (0.173 mL, 1.96 mmol, 1.1 equiv.) in CH_2_Cl_2_ (8.9 mL), boron trifluoride diethyl etherate (BF_3_·OEt_2_) (0.470 mL, 3.74 mmol, 2.1 equiv.) was added and the mixture was stirred at room temperature for 9 h. After completing the reaction, the solution was diluted with CH_2_Cl_2_, washed with aqueous 2% KOH solution (4 × 50 mL), dried over Na_2_SO_4_, and concentrated under reduced pressure. The crude product **1-V** (859 mg, >99% yield, yellow solid) was used in the next reaction without further purification.

Step 3:

*m*CPBA (assume 65%, 14.2 mmol, 8.0 equiv.) was dried in vacuo at room temperature for 1 h before the addition of CH_2_Cl_2_ (60 mL), and the solution was cooled to 0 °C. A solution of **1-V** (859 mg, 1.78 mmol, 1.0 equiv.) in CH_2_Cl_2_ (10 mL) was added, and the mixture was stirred at room temperature for 24 h. After completing the reaction, the solution was washed with aqueous 2% KOH solution (3 × 25 mL), dried over Na_2_SO_4_, and concentrated under reduced pressure. The crude product was purified by column chromatography on silica gel (*n*-hexane/CH_2_Cl_2_ = 1/4) to give product **1-VI** (895 mg, 80% yield for 3 steps) solid.

Several peak sets were observed due to the rotamers of the title compound. All peaks are given below:

mp = 322.4–333.5 °C (CHCl_3_); MS (ESI, *m*/*z*) 545 [(M − H)^−^]; HRMS (ESI) calcd. for C_33_H_22_NaO_4_S_2_ [(M + Na)^+^]: 569.0857 found 569.0863; ^1^H NMR (CDCl_3_, 500 MHz): δ = 4.59 (s, 2H), 7.09–7.11 (m 2H), 7.39–7.55 (m, 12H), 7.68–7.71 (m, 2H), 8.00 (d, *J* = 8.5 Hz, 2H), 8.06 (s, 2H); ^13^C NMR (CDCl_3_, 126 MHz): δ = 83.9, 127.1, 127.7, 127.8, 128.0, 128.29, 128.35, 130.1, 130.6, 131.3, 132.3, 133.8, 134.8, 138.1, 139.2, 139.5; IR (KBr) 506, 539, 650, 700, 728, 764, 779, 810, 828, 906, 1099, 1130, 1148, 1170, 1213, 1340, 1396, 1493, 1575, 2913, 2977, 3024, 3063 cm^−1^.

*(R)-4-Fluoro-2,6-diphenyl-4H-dinaphtho[2,1-d:1′,2′-f][1,3]dithiepine 3,3,5,5-tetraoxide* (**1-VII**)

To a suspension of NaH (60% oil suspension, 66.4 mg, 1.05 equiv.) in THF (1.5 mL) cooled in an ice/water bath, a solution of bis(phenylsulfonyl)methane **1-VI** (863 mg, 1.58 mmol, 1.0 equiv.) in THF (9.5 mL) was added portion-wise and the mixture was stirred for 30 min at room temperature. The resulting mixture was added to a suspension of selectfluor^®^ (588 mg, 1.66 mmol, 1.05 equiv.) in acetonitrile (MeCN) (3.3 mL) at 0 °C and the residue was rinsed with THF (5.0 mL). The mixture was allowed to warm to room temperature and stirred for 10 h. After completing the reaction, the solvents were removed under reduced pressure, and the residue was diluted with H_2_O and extracted with CH_2_Cl_2_. The combined organic layer was washed with brine, dried over anhydrous Na_2_SO_4_, and concentrated under reduced pressure. The crude product was purified by column chromatography on silica gel (*n*-hexane/CH_2_Cl_2_ = 2/3 to 1/9) to give product **1-VII** (613 mg, 69% yield) as a white solid.

Several peak sets were observed due to the rotamers of the title compound. All peaks are given below:

mp = 300.6–301.6 °C (CHCl_3_); MS (ESI, *m*/*z*) 587 [(M + Na)^+^]; HRMS (ESI) calcd. for C_33_H_21_FNaO_4_S_2_ [(M + Na)^+^]: 587.0763 found 587.0757; ^1^H NMR (CDCl_3_, 500 MHz): δ = 5.56 (d, *J* = 46.0 Hz, 2H), 7.11 (d, *J* = 8.8 Hz, 2H), 7.41–7.54 (m, 12H), 7.70–7.74 (m, 2H), 7.99–8.01 (m, 2H), 8.08 (d, *J* = 8.0 Hz, 2H), 8.06 (s, 2H); ^13^C NMR (CDCl_3_, 126 MHz): δ = 112.9 (d, *J* = 271.1 Hz), 127.0, 127.1, 127.75, 127.78, 127.80, 127.9, 128.1, 128.3, 128.41, 128.43, 128.45, 128.49, 128.6, 130.48, 130.50, 130.6, 131.2, 132.1, 132.2, 133.9, 134.0, 135.08, 135.12, 139.0, 139.06, 139.09, 139.4, 139.8, 141.0; ^19^F NMR (CDCl_3_, 282 MHz): δ = −173.2 (d, *J* = 46.0 Hz, 1F); IR (KBr) 531, 541, 650, 699, 730, 752, 769, 795, 905, 1097, 1128, 1146, 1173, 1189, 1353, 1396, 1445, 1492, 1574, 2923, 3028, 3055 cm^−1^.

*(R)-4-Fluoro-4-iodo-2,6-diphenyl-4H-dinaphtho[2,1-d:1′,2′-f][1,3]dithiepine 3,3,5,5-tetraoxide* ((R)**-1**)

To a solution of **1-VII** (595 mg, 1.05 mmol, 1.0 equiv.) in MeCN (7.9 mL), cesium carbonate (Cs_2_CO_3_) (411 mg, 1.26 mmol, 1.2 equiv.) was added and the mixture was stirred at room temperature for 15 min. Iodine (I_2_) (348 mg, 1.37 mmol, 1.3 equiv.) was added to the mixture and stirred at room temperature for 4 h. After completing the reaction, the solvent was removed under reduced pressure and the residue was dissolved in CH_2_Cl_2_/H_2_O. The aqueous layer was discarded, and the organic layer was washed with saturated aqueous Na_2_S_2_O_3_ solution and brine, dried over anhydrous Na_2_SO_4_, and concentrated under reduced pressure. The crude product was purified by column chromatography on silica gel (*n*-hexane/CH_2_Cl_2_ = 2/3) to afford halogenated product (*R*)-**1** (643 mg, 89% yield) as a slightly yellow solid.

mp = 217.8–218.7 °C (CHCl_3_); MS (ESI, *m*/*z*) 713 [(M + Na)^+^]; HRMS (ESI) calcd. for C_33_H_20_FINaO_4_S_2_ [(M + Na)^+^]: 712.9729 found 712.9725; ^1^H NMR (CDCl_3_, 500 MHz): δ = 7.12–7.20 (m 2H), 7.40–7.50 (m, 11H), 7.69–7.73 (m, 3H), 7.99 (d, *J* = 8.0 Hz, 2H), 8.04–8.06 (m, 2H); ^13^C NMR (CDCl_3_, 126 MHz): δ = 114.6 (d, *J* = 339.7 Hz), 125.7, 126.7, 126.8, 126.9, 127.76, 127.82, 127.9, 128.0, 128.1, 128.36, 128.44, 128.5, 128.6, 130.2, 130.5, 130.7, 131.6, 132.1, 132.2, 133.9, 134.1, 135.0, 135.2, 139.00, 139.02, 139.9, 140.19, 140.22, 141.4; ^19^F NMR (CDCl_3_, 282 MHz): δ = −112.7 (s, 1F); IR (KBr) 517, 532, 545, 577, 588, 630, 642, 700, 730, 762, 778, 904, 1138, 1158, 1170, 1356, 1395, 1444, 1492, 1573, 3024, 3055 cm^−1^.

### 3.3. Experimental Procedure of ^19^F NMR Titration of (R)-***1*** with nBu_4_NCl in CDCl_3_

To a solution of (*R*)-**1** (20.7 mg, 0.03 mmol) in CDCl_3_ (0.6 mL) in an NMR tube, a solution of *n*Bu_4_NCl (1.5 M in CDCl_3_) was added and ^19^F NMR of the mixture was taken when the added *n*Bu_4_NCl solution was 0, 0.5, 1.0, and 2.0, equivalent to (*R*)-**1**. Chemical shifts (δ) are recorded in ppm downfield from C_6_F_6_ (δ = −162.2 (CDCl_3_)) as an internal standard.

### 3.4. General Procedure of Mukaiyama Aldol with (R)-***1***

Silyl ketene acetals **3** were prepared according to published literature [28].

To a solution of (*R*)-**1** (3.5 mg, 0.005 mmol, 10 mol%) and aldehyde **2** (0.05 mmol, 1.0 equiv.) in CH_2_Cl_2_ (0.05 mL), silyl ketene acetal **3** (0.075 mmol, 1.5 equiv.) was added and the mixture was stirred at room temperature for 12 h. After completing the reaction, the mixture was filtered through a pad of silica gel, and the filtrate was concentrated under reduced pressure. The crude product was purified by column chromatography on silica gel to give the desired product **4**. Next, to a solution of **4** in THF (0.25 mL), tetrabutylammonium fluoride (TBAF) (1.0 M in THF, 1.0 equiv.) was added and the mixture was stirred at room temperature for 1 h. The crude product was purified by column chromatography on silica gel to give desilylated product **5**.

*Methyl 3-((tert-butyldimethylsilyl)oxy)-3-(naphthalen-2-yl)propanoate* (**4b**)

Following the general procedure, (*R*)-**1** (3.5 mg, 0.005 mmol, 10 mol%), aldehyde **2** (7.8 mg, 0.05 mmol, 1.0 equiv.), and silyl ketene acetal **3b** (14.1 mg, 0.0075 mmol, 1.5 equiv.) were used in CH_2_Cl_2_ (0.05 mL) at room temperature for 12 h. The crude product was purified by column chromatography on silica gel (*n*-hexane/EtOAc = 97/3) to give product **4b** (13.3 mg, 77% yield) as a white solid.

The ^1^H NMR spectrum matched the one reported by B. List et al. [29].

MS (ESI, *m*/*z*) 367 [(M + Na)^+^]; ^1^H NMR (CDCl_3_, 300 MHz): δ = −0.17 (s, 3H), 0.04 (s, 3H), 0.86 (s, 9H), 2.63 (dd, *J* = 4.1, 14.7 Hz, 1H), 2.82 (dd, *J* = 9.3, 14.7 Hz, 1H), 3.69 (s, 3H), 5.33 (dd, *J* = 4.1, 9.3 Hz, 1H), 7.46–7.52 (m, 3H), 7.77–7.84 (m, 4H).

*Methyl 3-hydroxy-3-(naphthalen-2-yl)propanoate* (**5**)

The ^1^H NMR spectrum matched the one reported by Denmark et al. [30].

MS (ESI, *m*/*z*) 253 [(M + Na)^+^]; ^1^H NMR (CDCl_3_, 300 MHz): δ = 2.78–2.88 (m, 2H), 3.32–3.33 (d, *J* = 2.7 Hz, 1H), 3.73 (s, 3H), 5.30–5.32 (m, 1H), 7.44–7.51 (m, 3H), 7.83–7.84 (m, 4H).

### 3.5. General Procedure of Reduction of Quinoline with (R)-***1***

Quinoline **6** was prepared according to published literature [31].

To a mixture of (*R*)-**1** (6.9 mg, 0.01 mmol, 10 mol%), quinoline **6** (20.5 mg, 0.1 mmol, 1.0 equiv.) and Hantzsch ester **7** (55.7 mg, 0.220 mmol, 2.2 equiv.) were added in CH_2_Cl_2_ (1.4 mL) at room temperature for 24 h. The crude product was purified by column chromatography on silica gel (*n*-hexane/EtOAc = 97/3) to give product **8** (20.5 mg, 98% yield) as a yellow oil.

The ^1^H NMR spectrum matched the one reported by Lacôte et al. [32].

MS (ESI, *m*/*z*) 210 [(M + H)^+^]; ^1^H NMR (CDCl_3_, 300 MHz): δ = 1.93–2.15 (m, 2H), 2.69–2.78 (m, 1H), 2.87–2.98 (m, 1H), 4.03 (brs, 1H), 4.44 (dd, *J* = 3.0, 9.0 Hz, 1H), 6.53 (d, *J* = 7.5 Hz, 1H), 6.65 (t, *J* = 7.5 Hz, 1H), 7.01 (t, *J* = 7.2 Hz, 2H), 7.25–7.40 (m, 5H).

## 4. Conclusions

In summary, we have described the synthesis of a new chiral halogen-bond donor, which has a fluorobissulfonyl scaffold with a chiral binaphthyl backbone, and its application as a Lewis acid catalyst. A chiral halogen-bond donor (*R*)-**1** was synthesized from commercially available (*R*)-1,1′-binaphthol in 16% total yield in 11 steps. The use of a catalytic amount of (*R*)-**1** in the Mukaiyama aldol reaction and quinoline reduction afforded the products in high yield. As far as we know, these are the first examples of chiral XB catalysts containing a sp^3^-hybridized carbon–iodine moiety. While enantioselectivity could not be induced by (*R*)-**1** in the attempted reactions, the results should be useful for the new design of effective chiral XB catalysts containing a sp^3^-hybridized carbon–iodine moiety, such as bidentate-type catalysts, but not monodentate-type catalysts, as one example. Further investigation into these possibilities is ongoing in our laboratory.

## Supplementary Materials

The Supplementary Materials are available online, that contains ^1^H, ^13^C, and ^19^F-NMR spectra or IR spectra of **1**, **4**, **5** and **8**. Crystallographic data of (*R*)-**1** is also available from the Cambridge Crystallographic Database as file numbers CCDC 2031026.
