# Supplementary material for: Design and Synthesis of a Chiral Halogen-Bond Donor with a Sp3-Hybridized Carbon–Iodine Moiety in a Chiral Fluorobissulfonyl Scaffold
Source: Molecules. 2020 Oct 3;25(19):4539. doi: 10.3390/molecules25194539 (PMC7583727; doi:10.3390/molecules25194539)
Supplement: Supplementary file 1 [file molecules-25-04539-s001.pdf]

Supplementary Materials

Design and Synthesis of a Chiral Halogen-bond Donor with an sp^3-Hybridized Carbon–Iodine Moiety in a Chiral Fluorobissulfonyl Scaffold

Hiroto Uno,[1] Kohei Matsuzaki,[1] Motoo Shiro,[2] and Norio Shibata [1,3,*]

[1] Department of Nanopharmaceutical Sciences, and Department of Life Science and Applied Chemistry, Nagoya Institute of Technology, Gokiso, Showa-ku, Nagoya 466-8555, Japan

[2] Rigaku Corporation, 3-9-12, Matsubara cho, Akishima shi, Tokyo 196 8666, Japan.

[3] Institute of Advanced Fluorine-Containing Materials, Zhejiang Normal University, 688 Yingbin Avenue, 321004 Jinhua, China.

*E-mail: nozshiba@nitech.ac.jp

CONTENTS

^{1}H, ^{13}C and ^{19}F NMR spectra......1
IR spectra......17

^{1}H, ^{13}C and ^{19}F NMR spectra

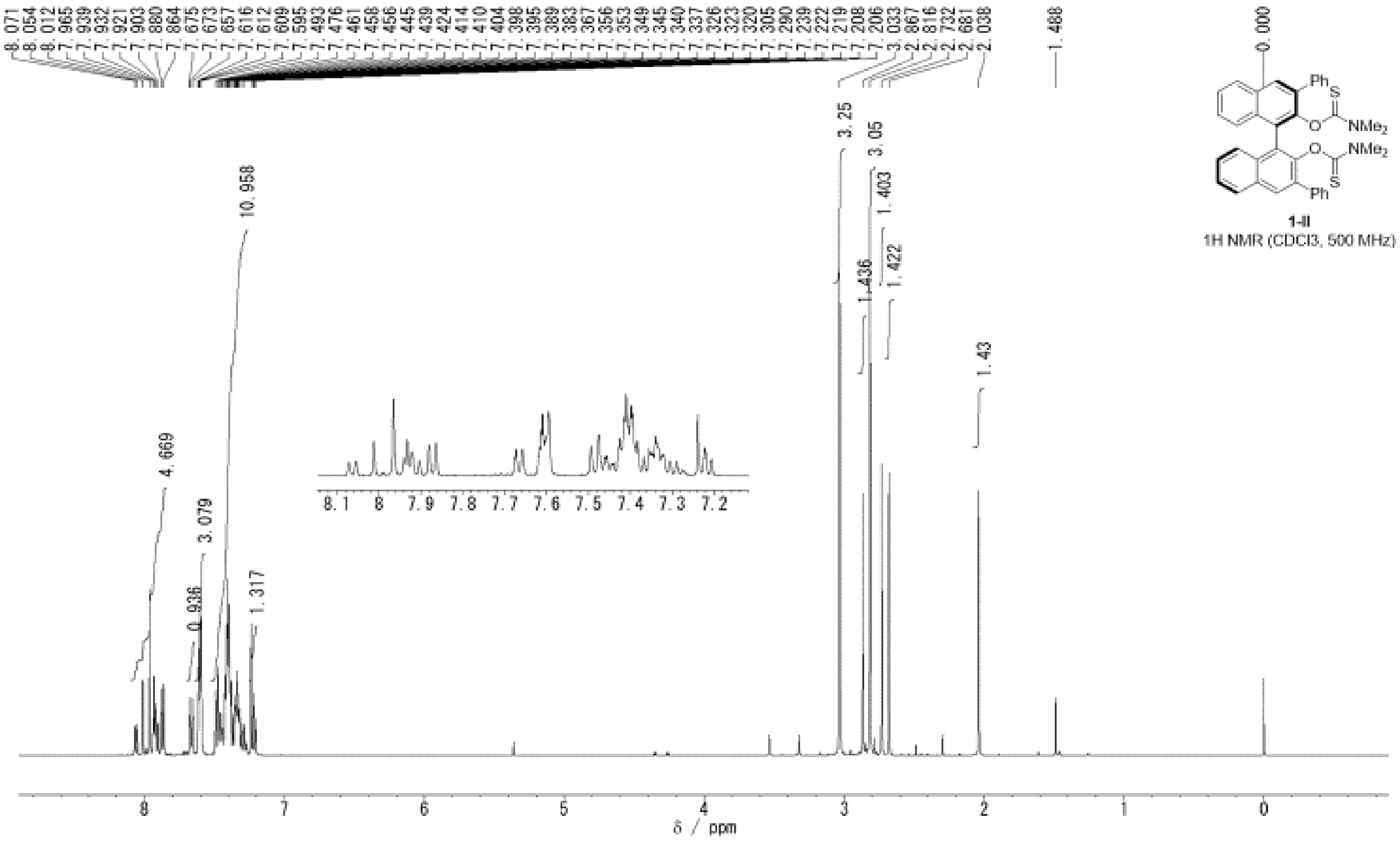

1-ll
1H NMR (CDCl3, 500 MHz)

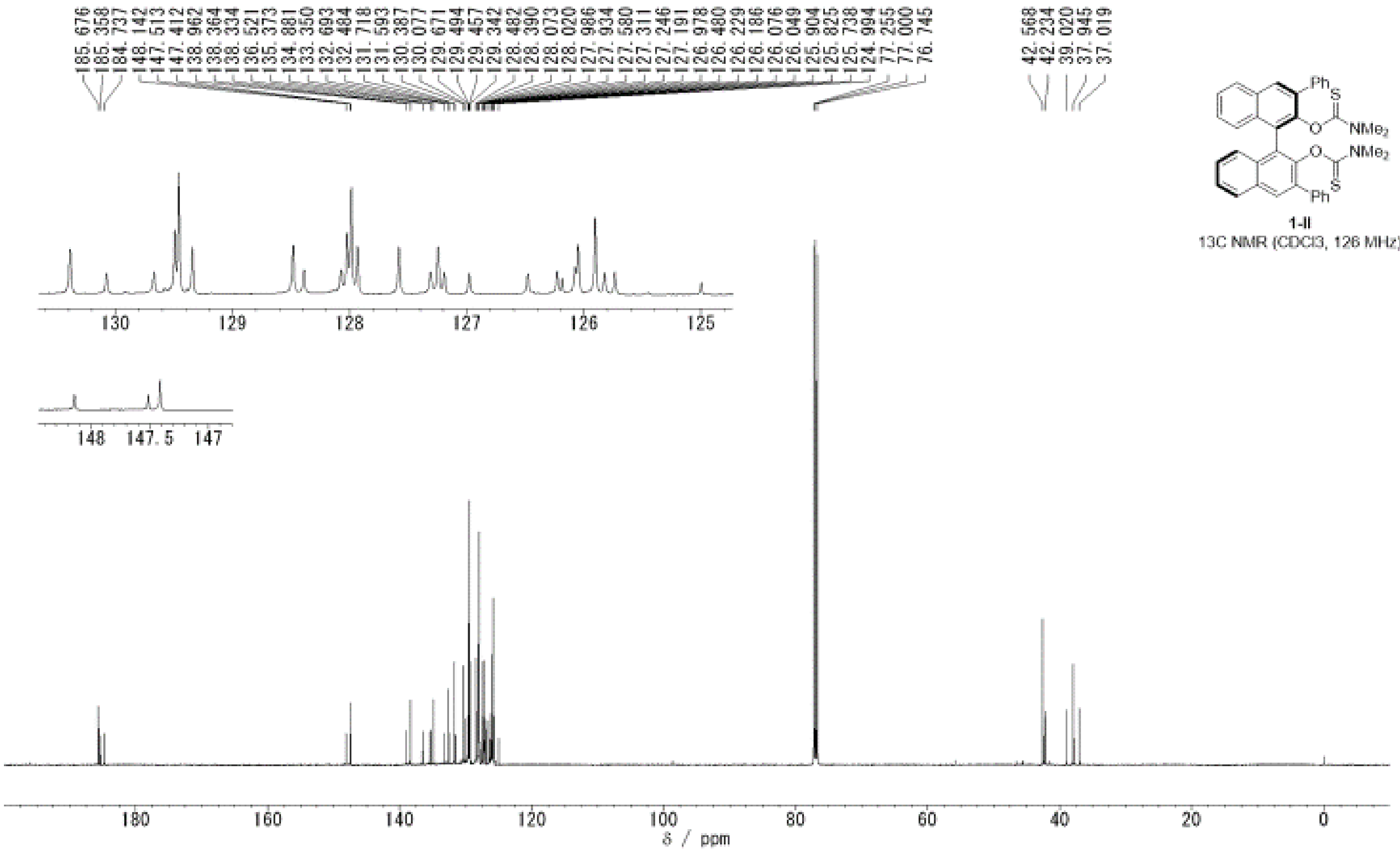

1-II

13C NMR (CDCl3, 126 MHz)

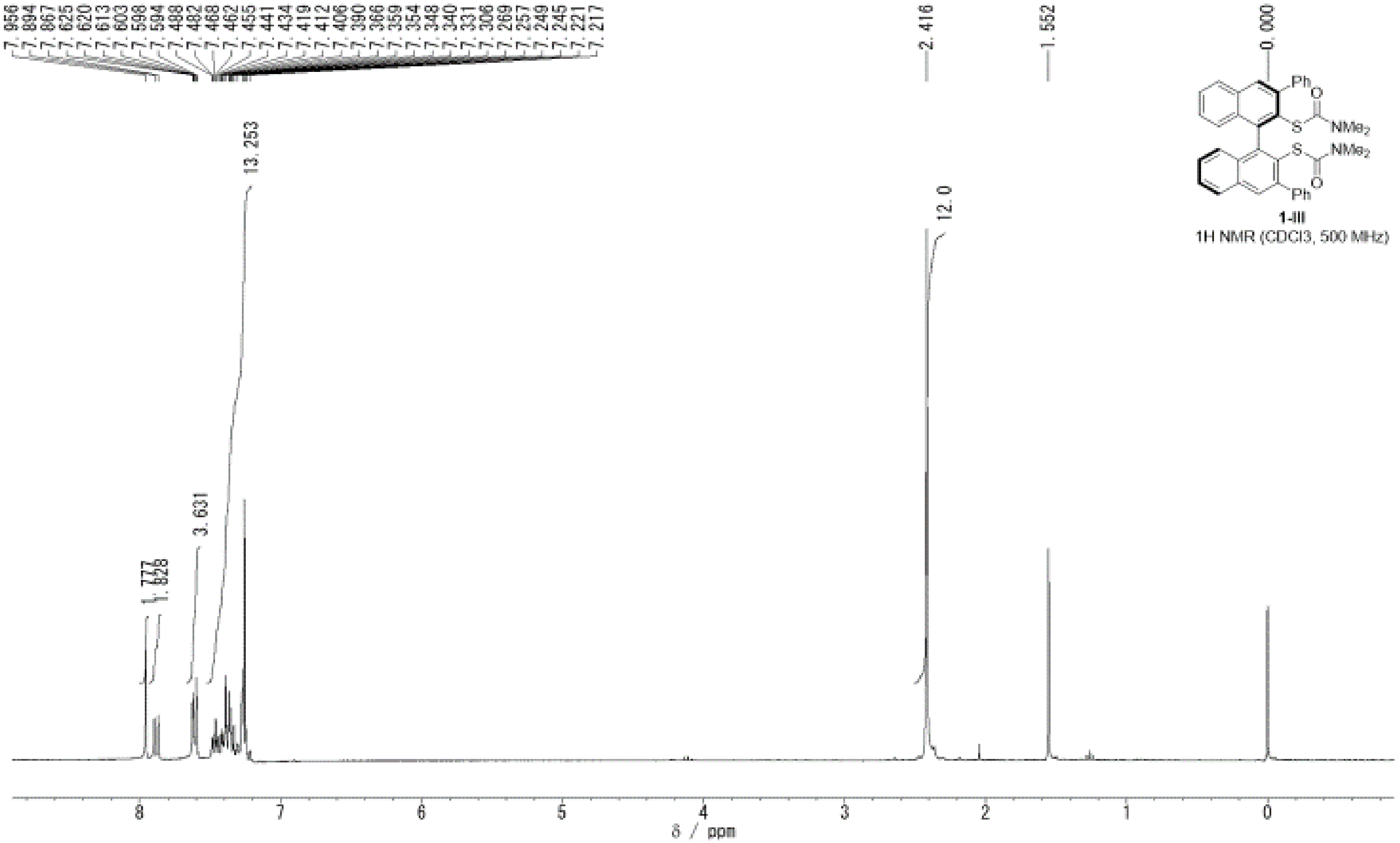
7.956
7.894
7.867
7.625
7.620
7.613
7.603
7.598
7.594
7.488
7.482
7.468
7.462
7.455
7.441
7.434
7.419
7.412
7.406
7.390
7.366
7.359
7.354
7.348
7.340
7.331
7.306
7.269
7.257
7.249
7.245
7.221
7.217
2.416
1.552
0.000
13.253
3.631
1.777
1.828
12.0
Ph
O
S
NMe2
S
NMe2
O
Ph
1-III
1H NMR (CDCl3, 500 MHz)
8
7
6
5
4
3
2
1
0
δ / ppm

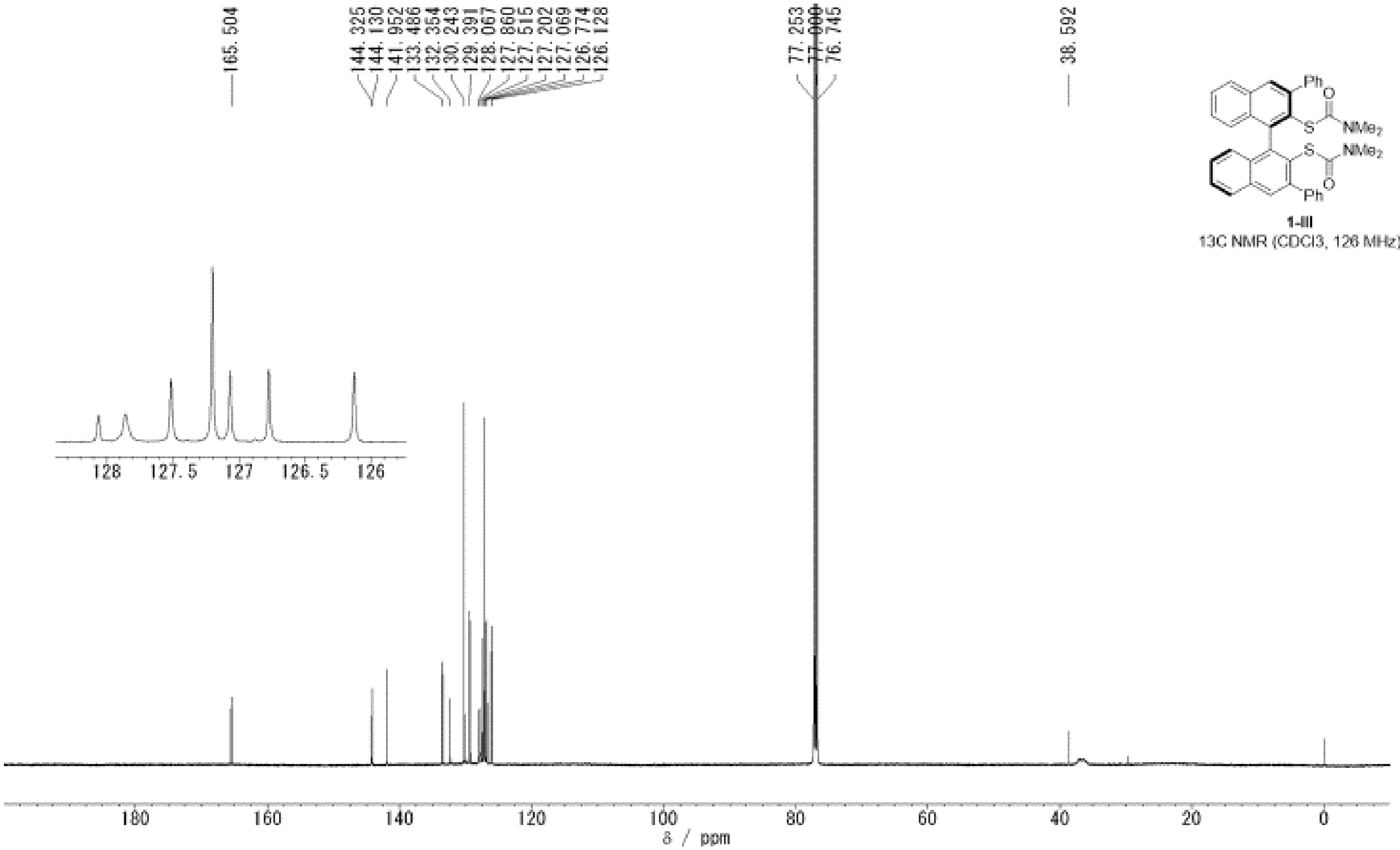

1-III

13C NMR (CDCl3, 126 MHz)

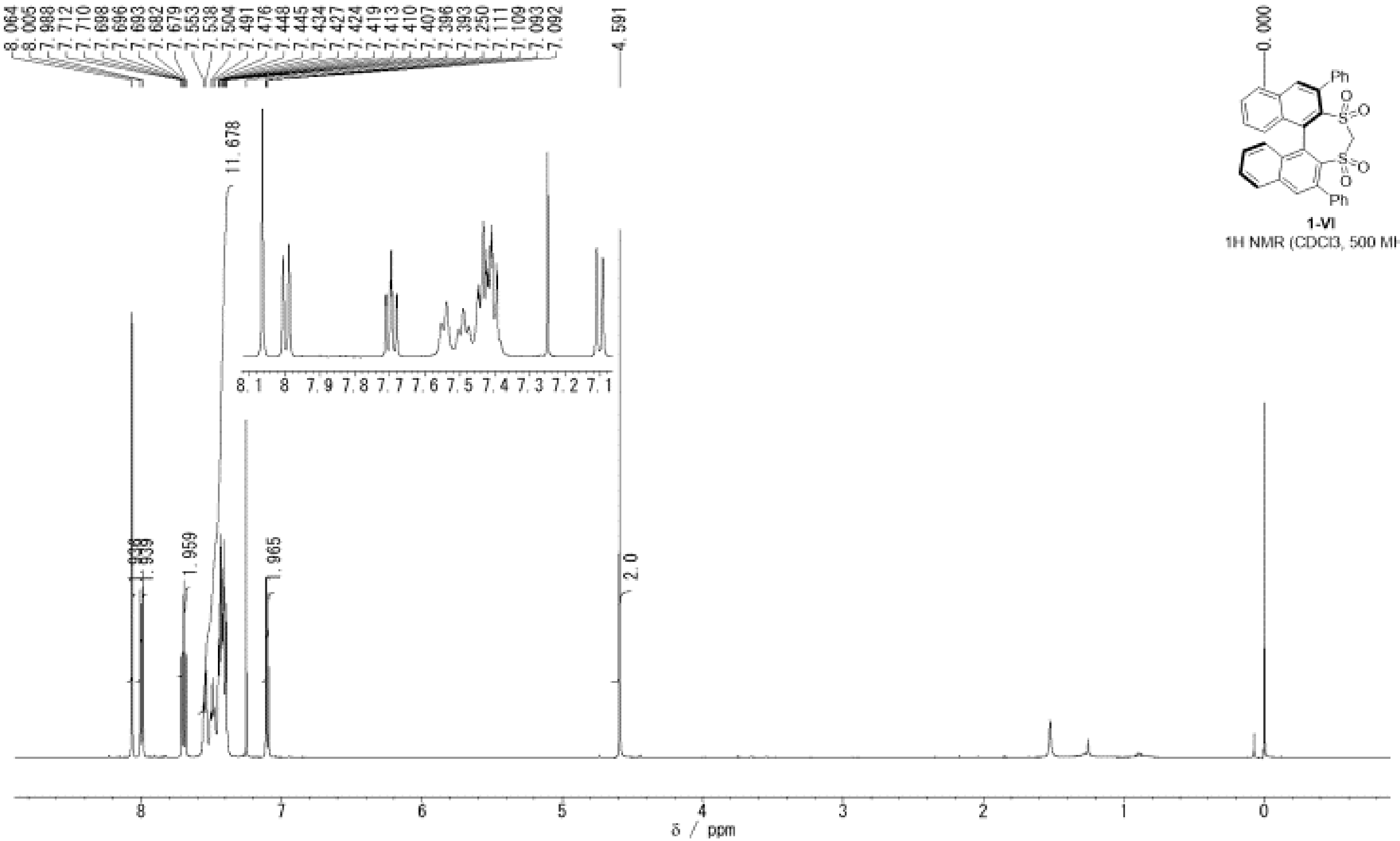

1-VI
1H NMR (CDCl3, 500 MHz)

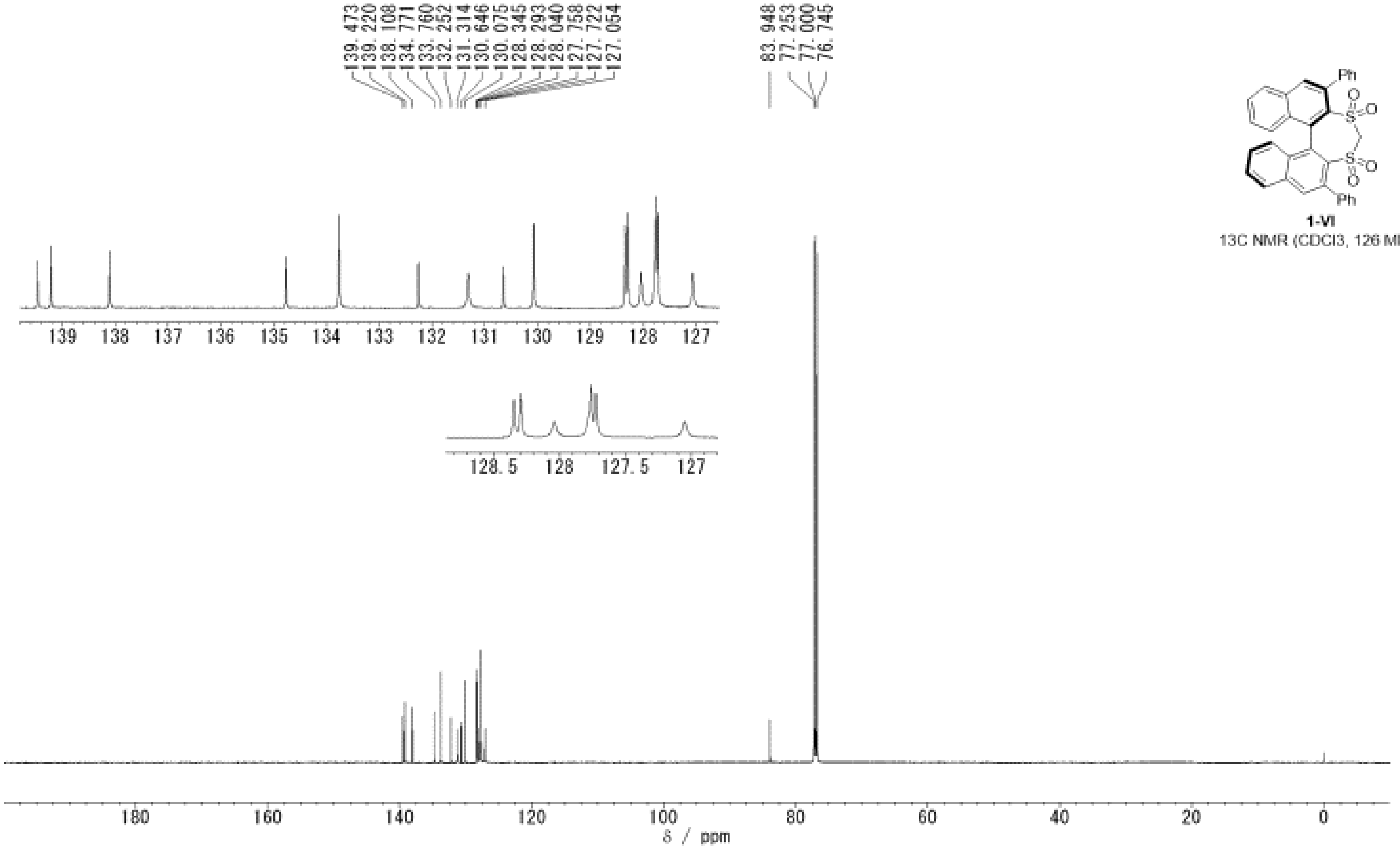

1-VI

13C NMR (CDCl3, 126 MHz)

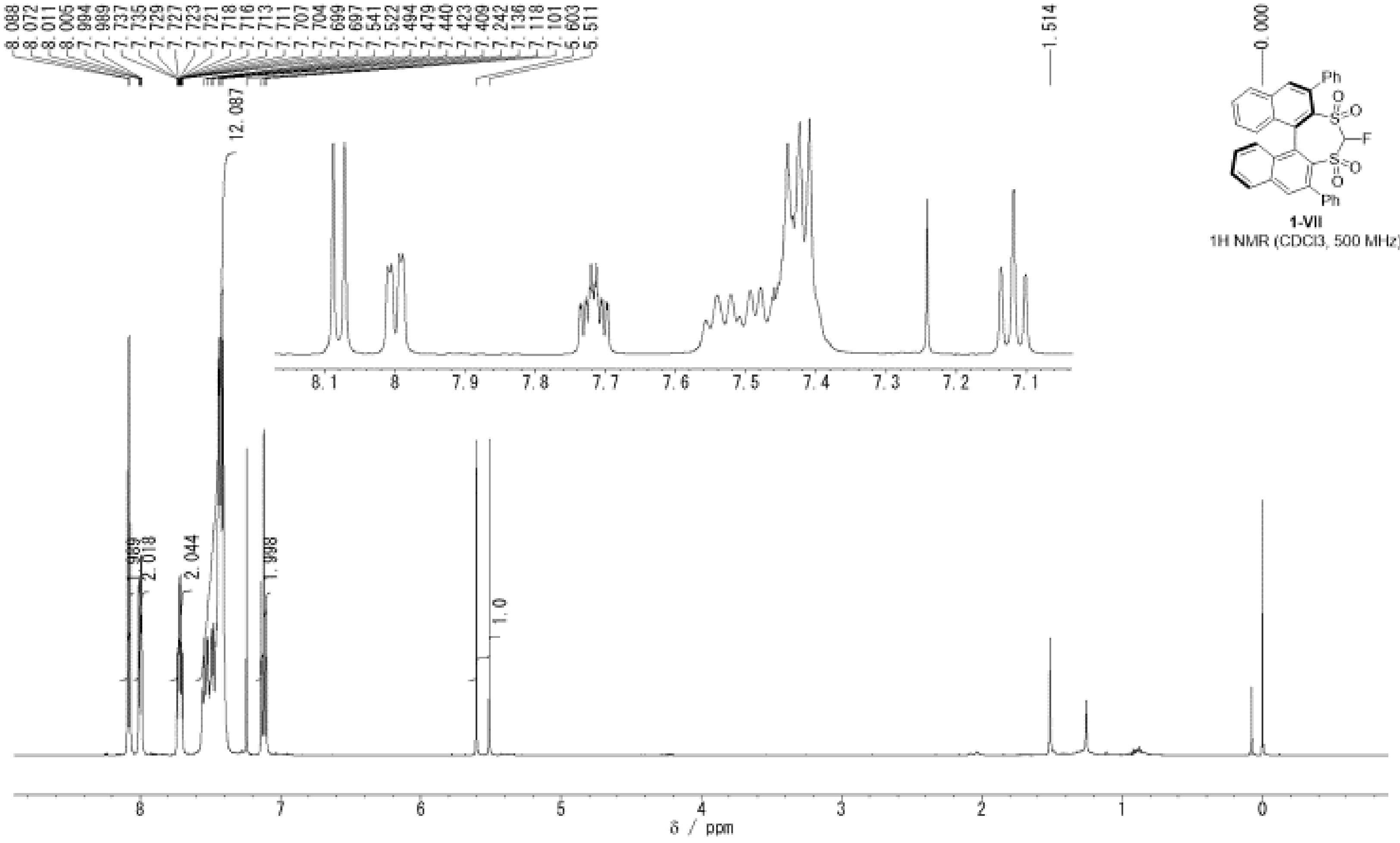

1-VII

1H NMR (CDCl3, 500 MHz)

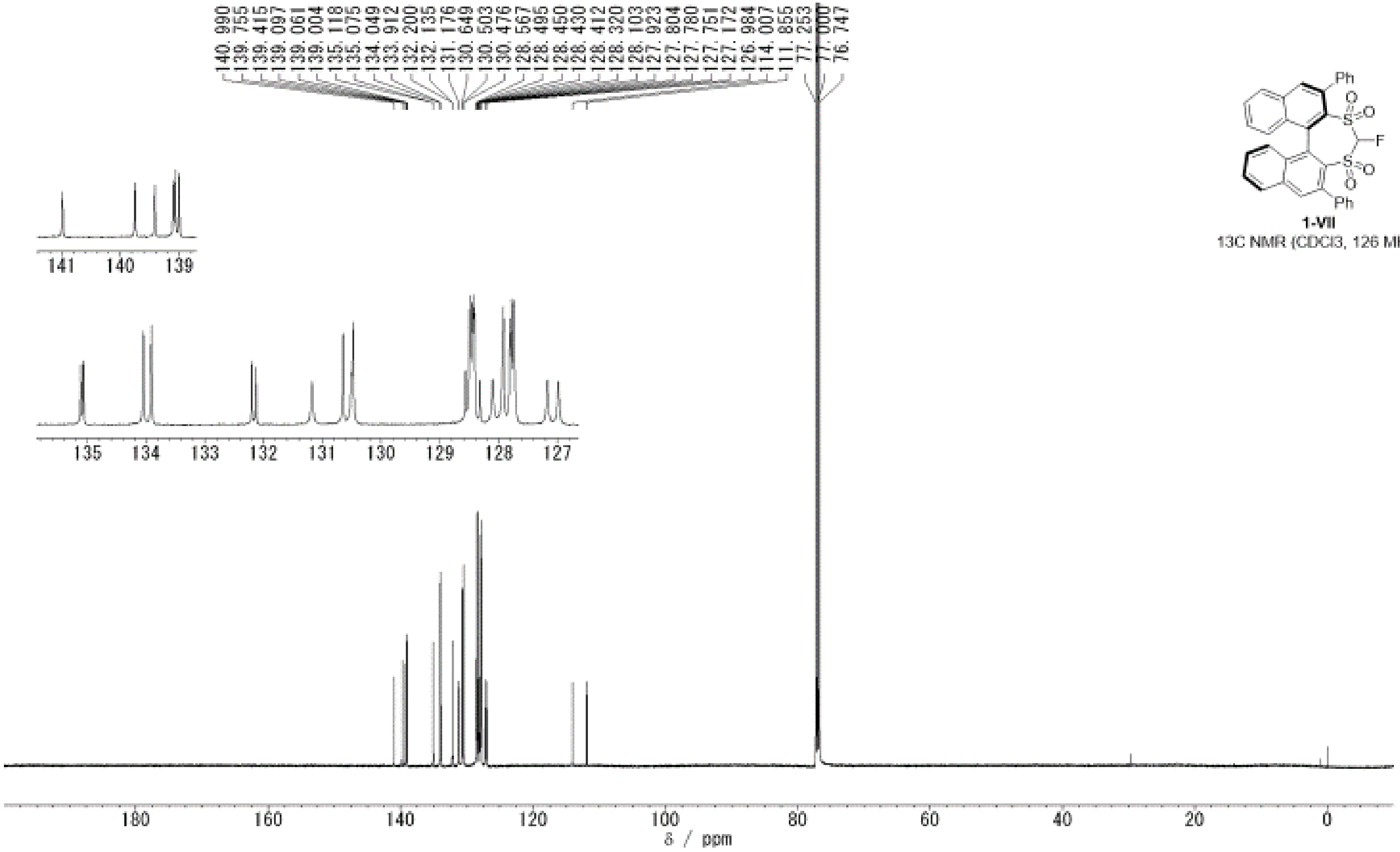

1-VII

13C NMR (CDCl3, 126 MHz)

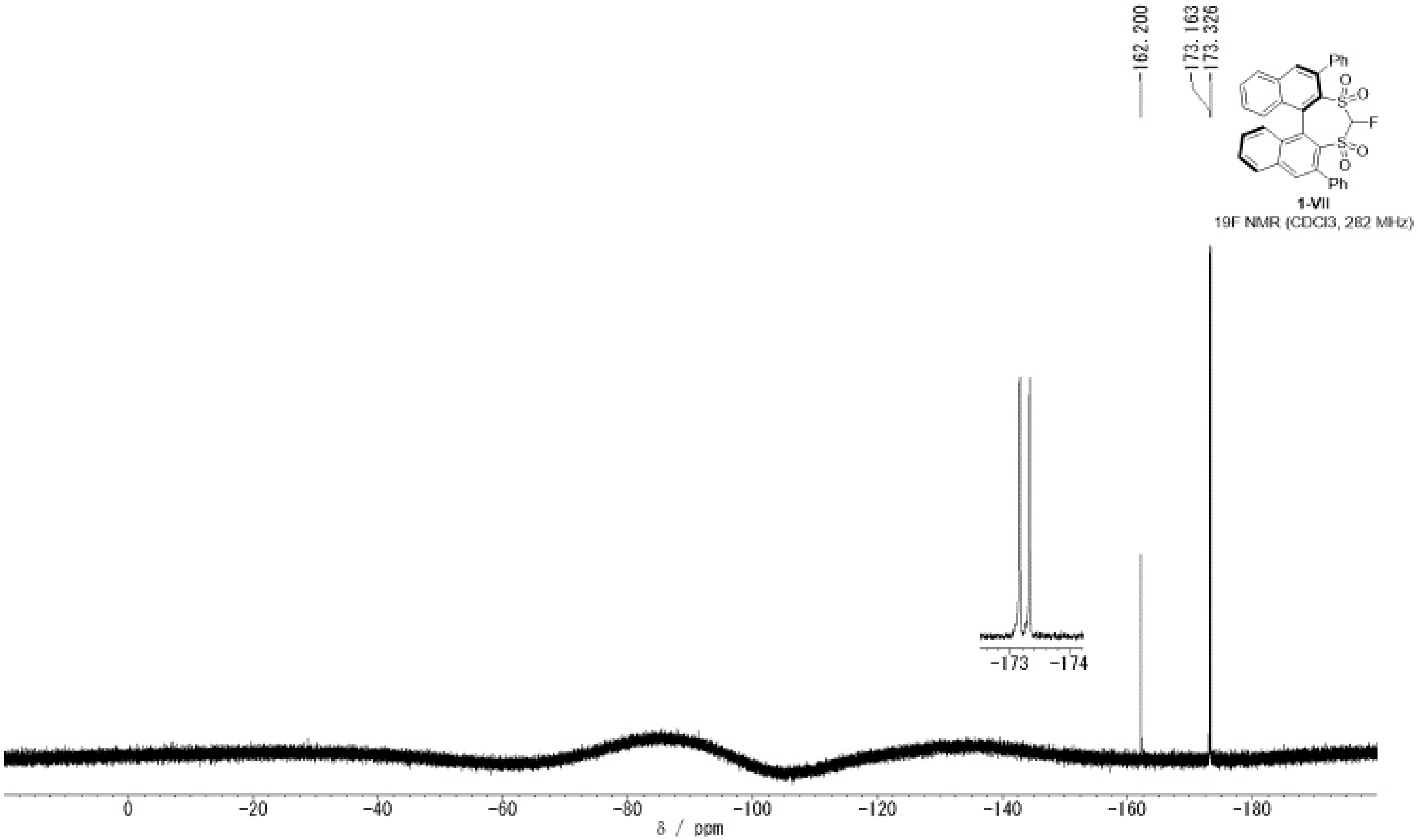

-162.200
-173.163
-173.326
Ph
S
O
O
F
Ph
1-VII
19F NMR (CDCl3, 282 MHz)
-173
-174
0
-20
-40
-60
-80
-100
-120
-140
-160
-180
δ / ppm

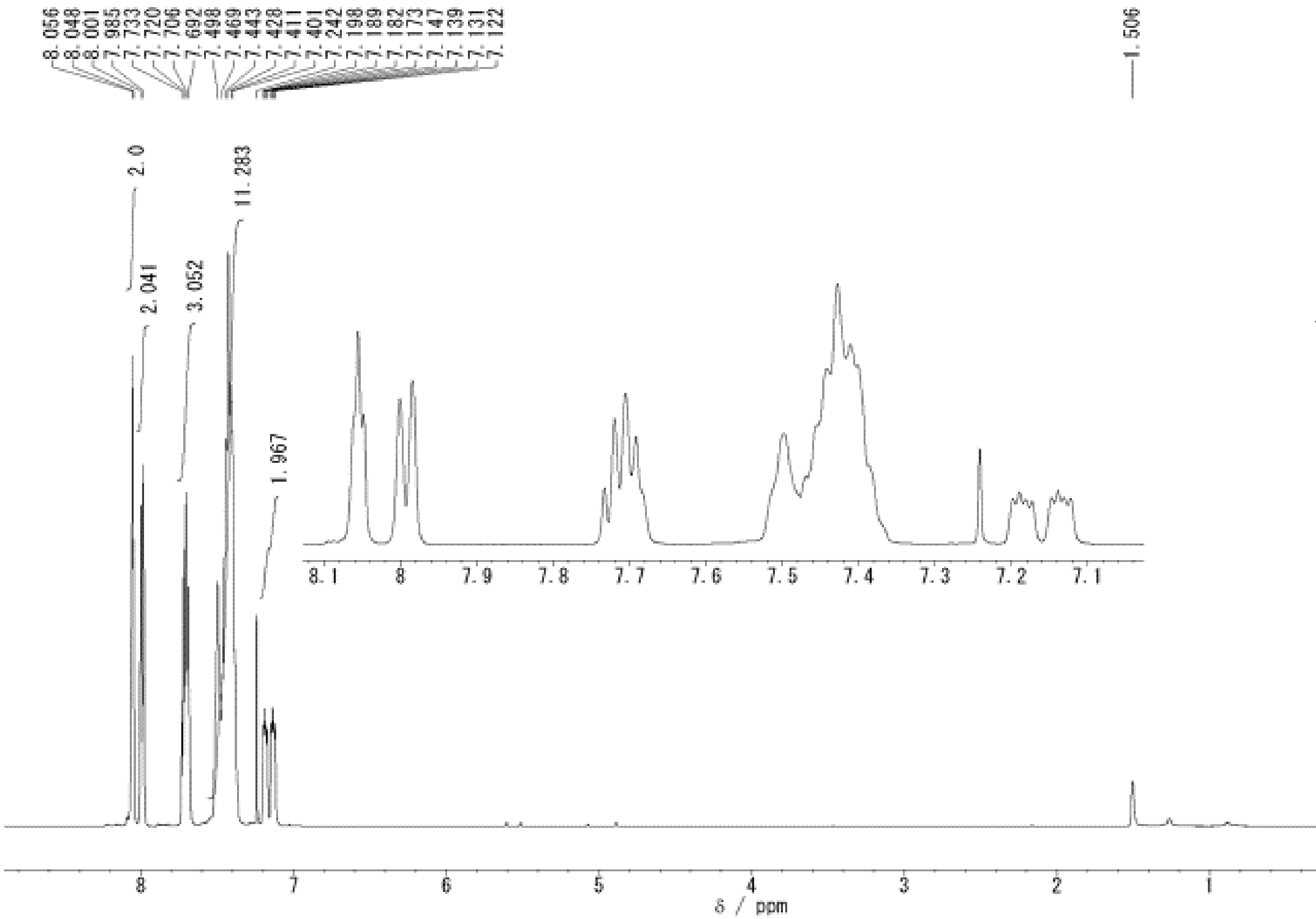

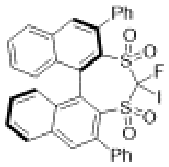

(*R*)-**1**
1H NMR (CDCl3, 500 MHz):

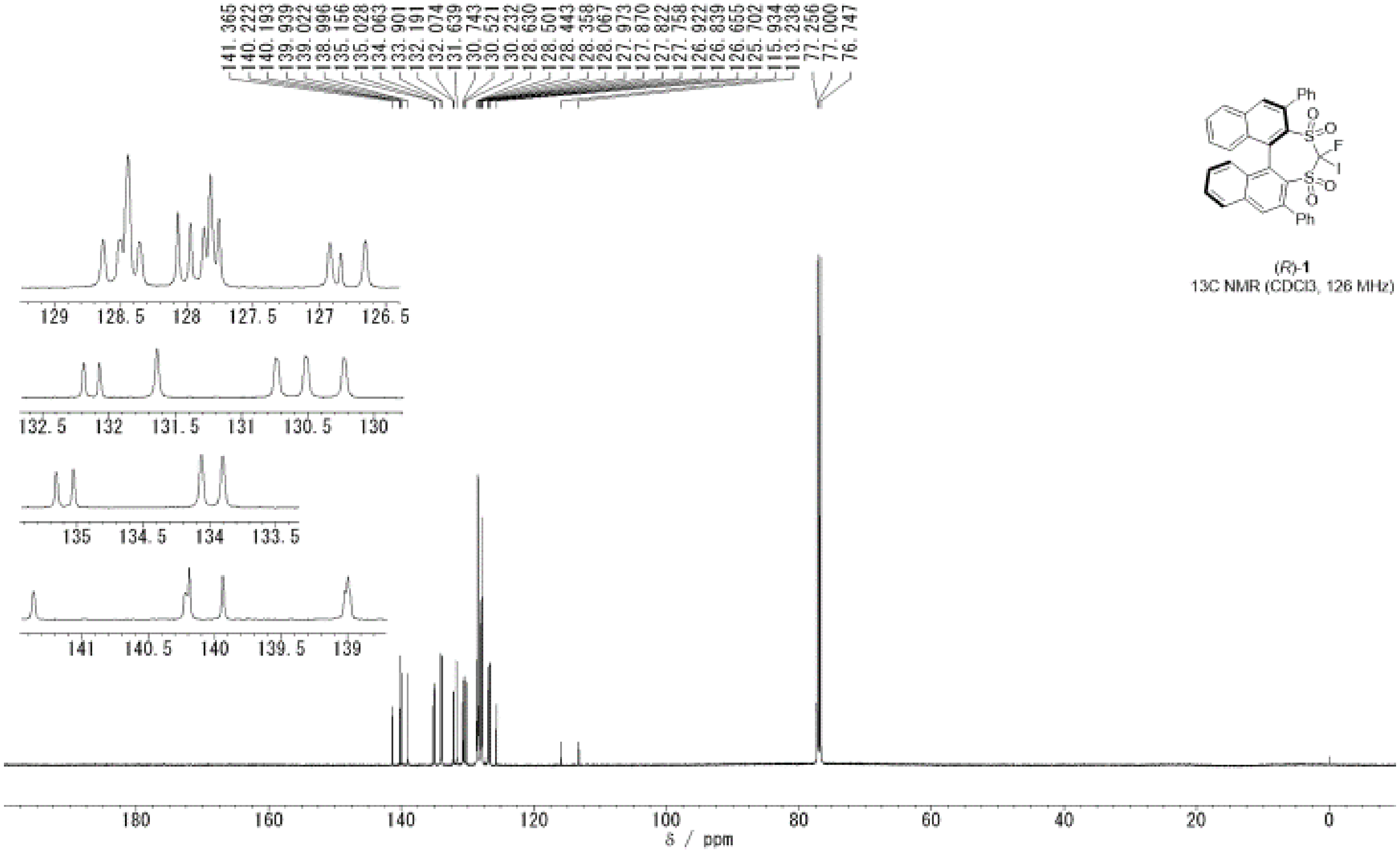

(R)-**1**

13C NMR (CDCl3, 126 MHz)

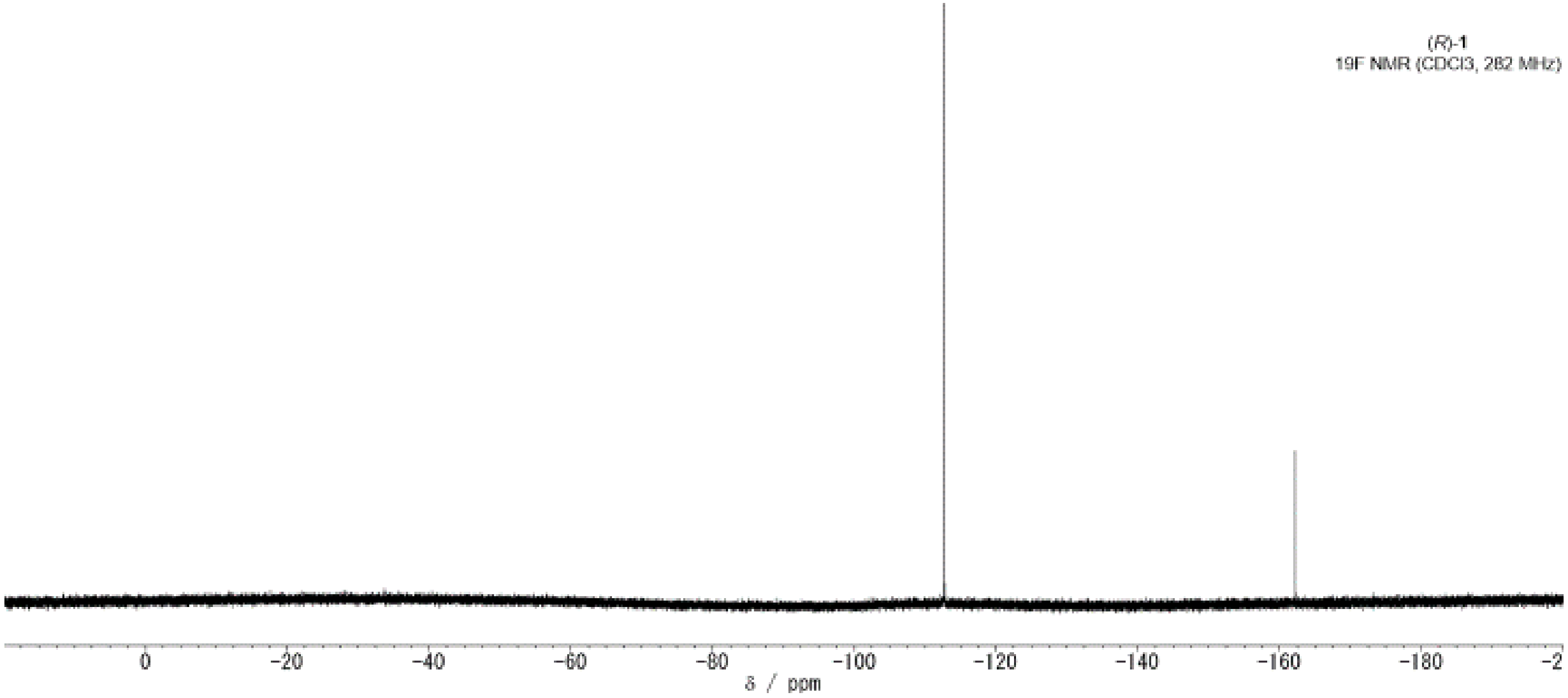

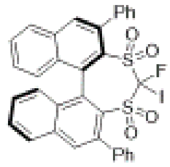

(*R*)-**1**
19F NMR (CDCl3, 282 MHz)

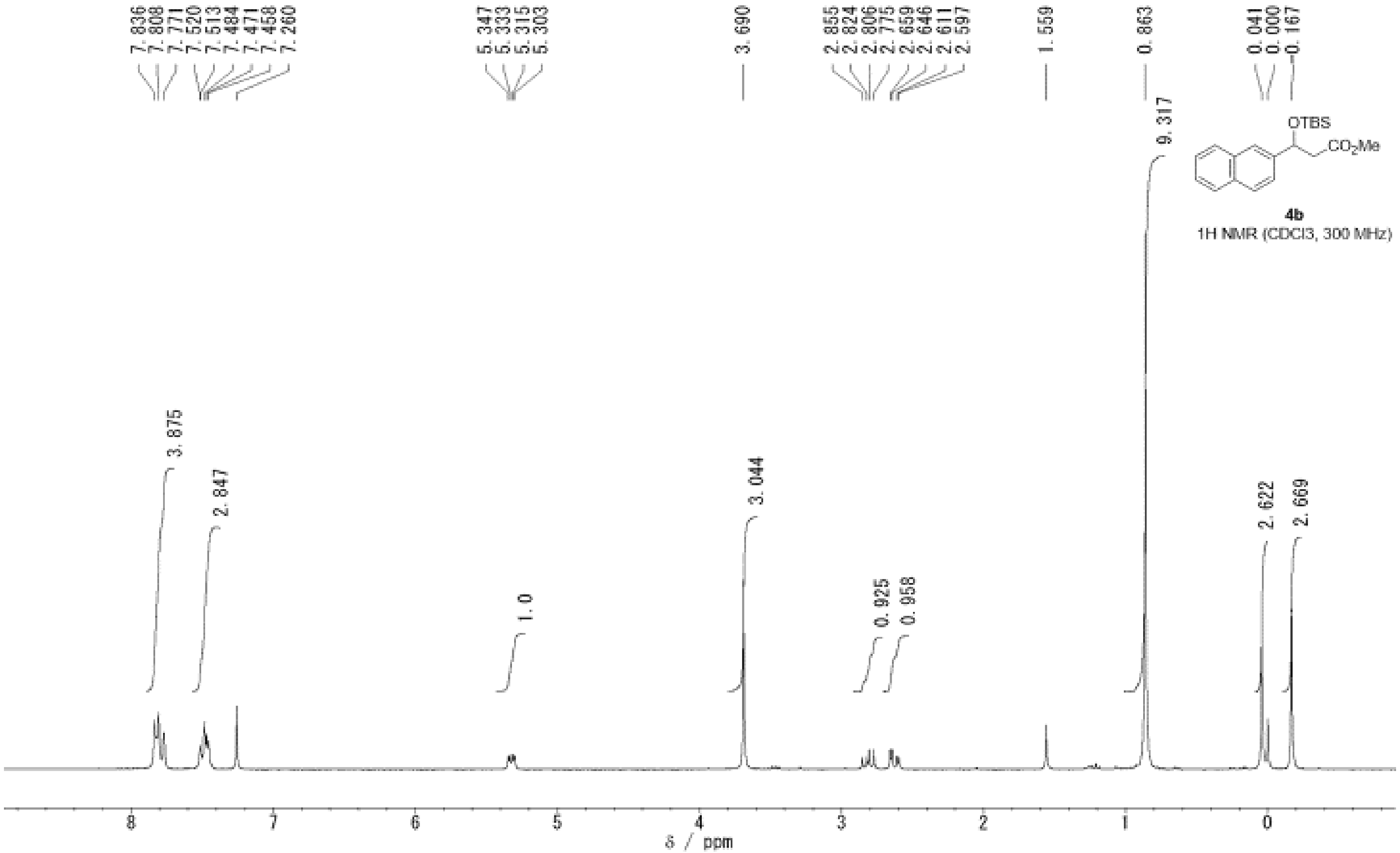

4b

1H NMR (CDCl3, 300 MHz)

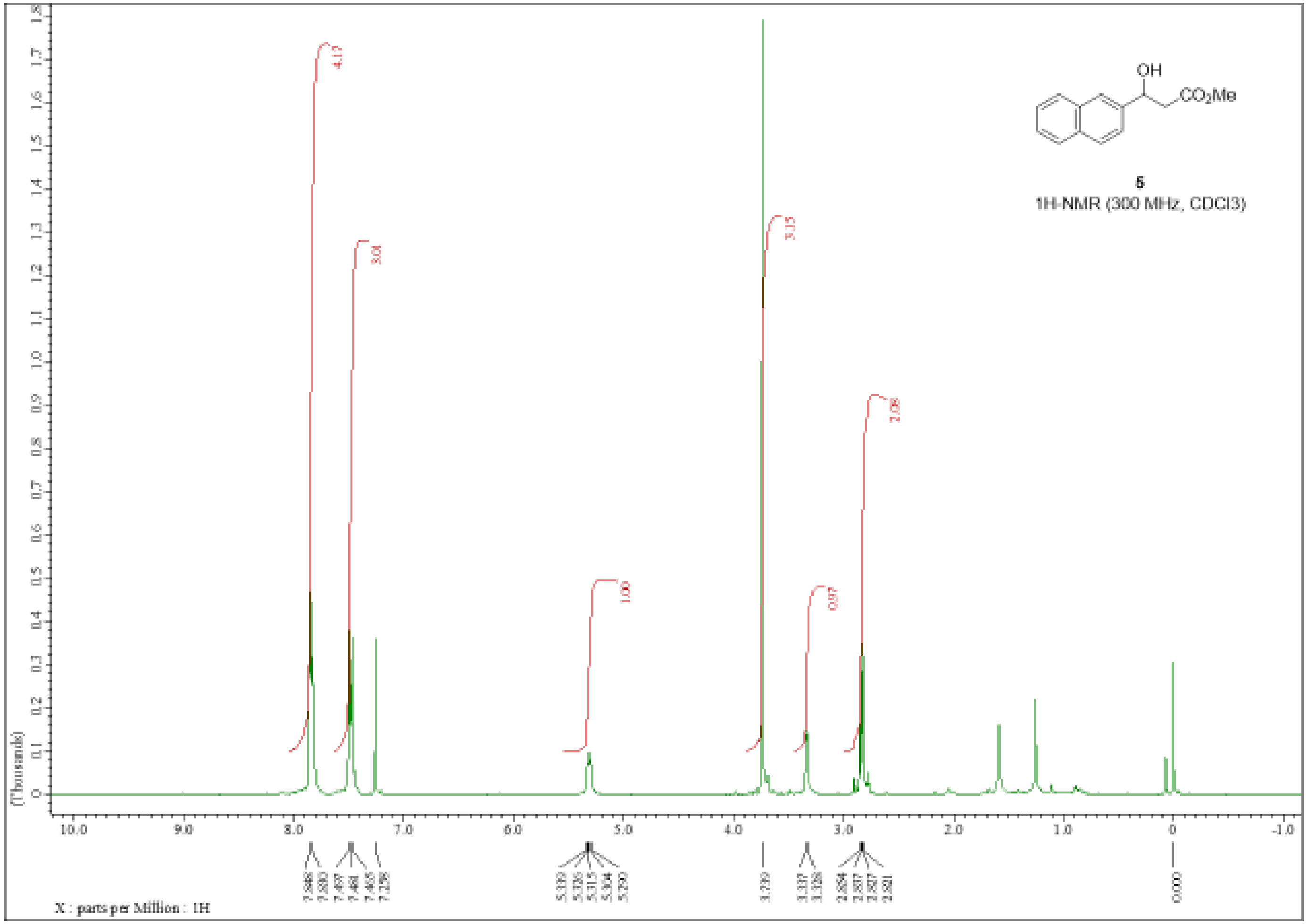

OH
CO2Me
5
1H-NMR (300 MHz, CDCl3)
X : parts per Million : 1H
(Thousands)

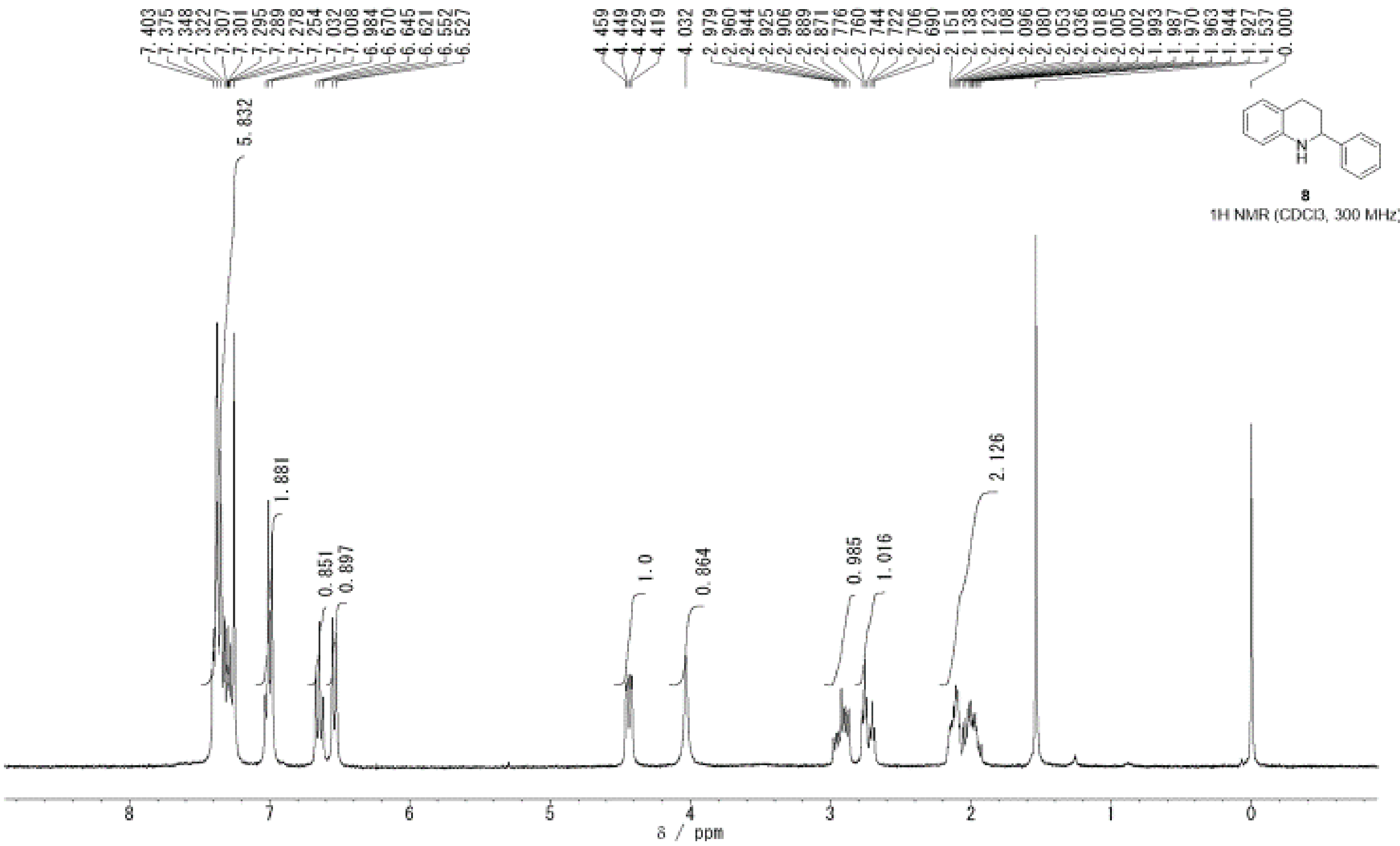

8

1H NMR (CDCl3, 300 MHz)

IR spectra

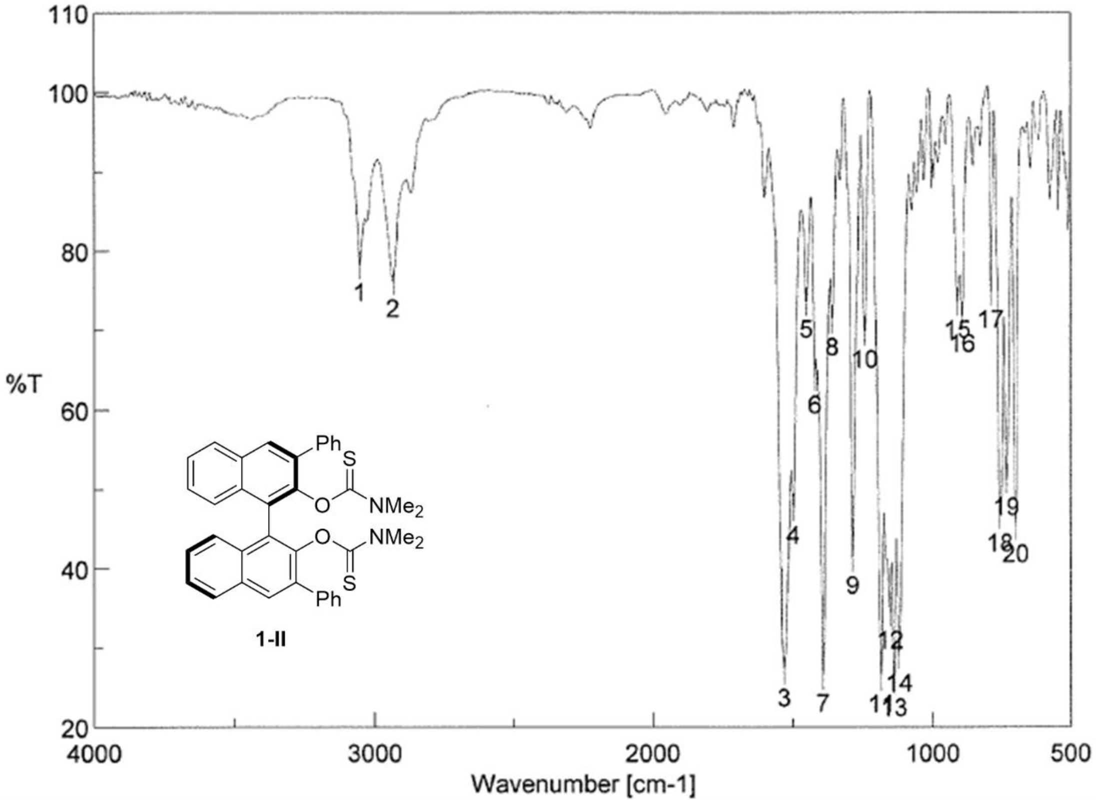

[information]

sample : 1-II

comment : KBr

[observed peaks]

No.	Position	Strength	No.	Position	Strength
1	3053.73	78.218	2	2935.13	76.184
3	1530.24	27.165	4	1497.45	47.658
5	1451.17	73.589	6	1420.32	64.059
7	1392.35	26.475	8	1359.57	71.341
9	1286.29	41.363	10	1243.86	69.809
11	1184.08	26.377	12	1152.26	34.468
13	1137.8	25.915	14	1119.48	29.030
15	910.236	73.527	16	892.88	71.760
17	789.707	74.805	18	756.923	46.640
19	732.817	51.226	20	699.069	45.321

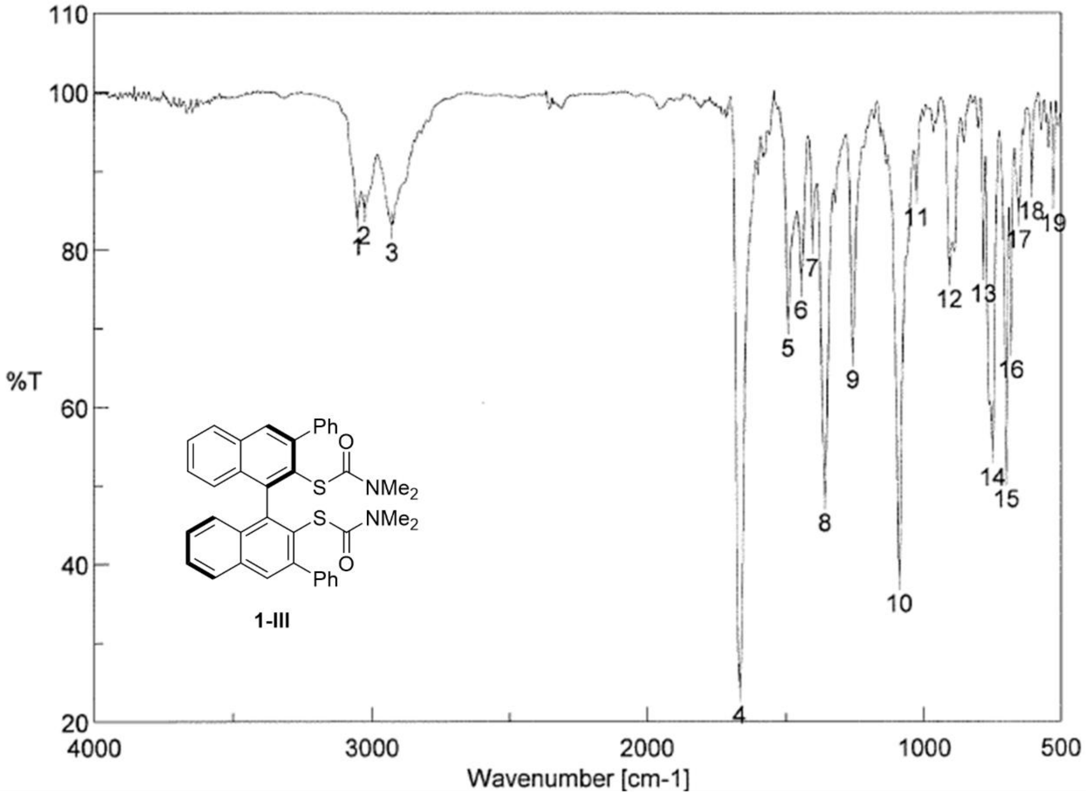

[information]
sample : 1-III
comment : KBr

[observed peaks]

No.	Position	Strength	No.	Position	Strength
1	3052.76	83.891	2	3026.73	85.292
3	2927.41	83.161	4	1666.2	24.326
5	1492.63	70.962	6	1444.42	75.824
7	1403.92	81.247	8	1359.57	48.715
9	1259.29	66.927	10	1090.55	38.472
11	1027.87	87.594	12	907.344	77.230
13	783.922	77.922	14	751.138	54.476
15	700.034	51.752	16	682.677	68.177
17	653.75	84.786	18	606.503	88.409
19	527.436	86.870			

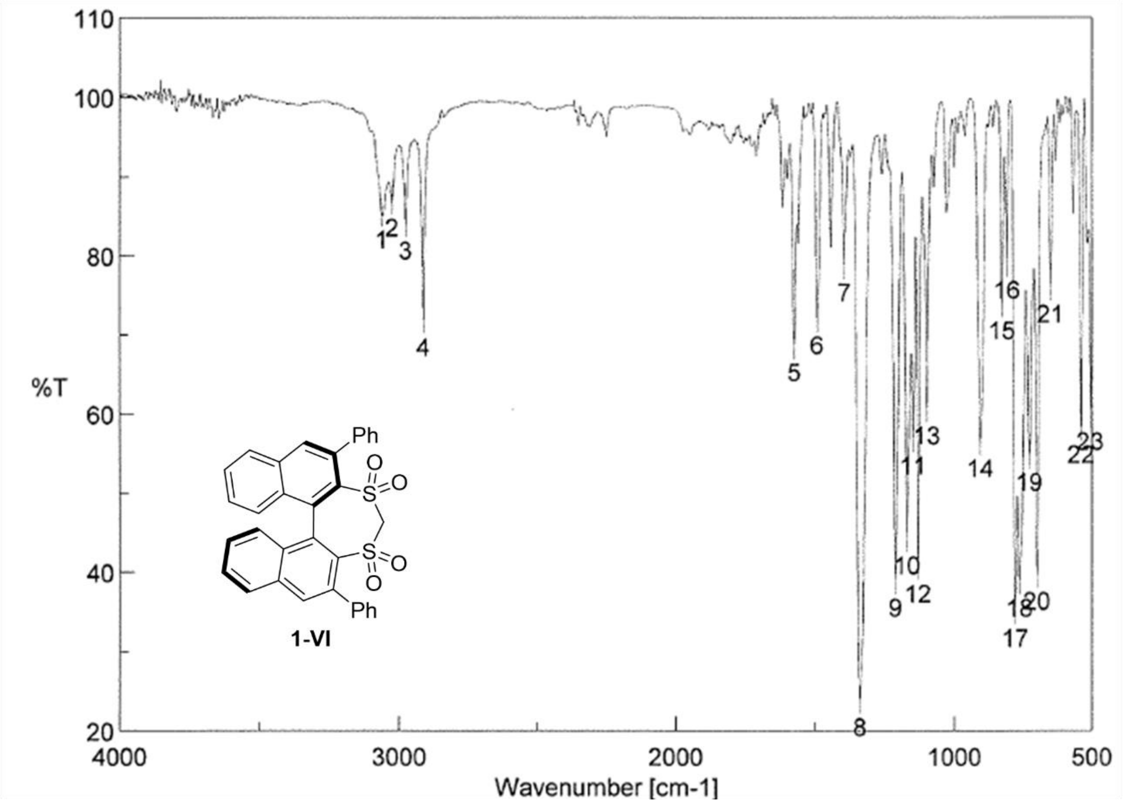

[information]
sample : 1-VI
comment : KBr

[observed peaks]

No.	Position	Strength	No.	Position	Strength
1	3063.37	85.476	2	3024.8	86.971
3	2977.55	84.091	4	2913.91	71.866
5	1575.56	68.682	6	1493.6	72.114
7	1396.21	78.826	8	1340.28	23.930
9	1213.01	38.927	10	1170.58	44.244
11	1148.4	56.879	12	1130.08	40.717
13	1099.23	60.691	14	906.379	56.524
15	828.277	73.964	16	810.92	79.129
17	779.101	35.127	18	764.637	38.890
19	728.961	54.786	20	700.034	39.756
21	650.858	76.056	22	539.971	58.289
23	506.223	59.931			

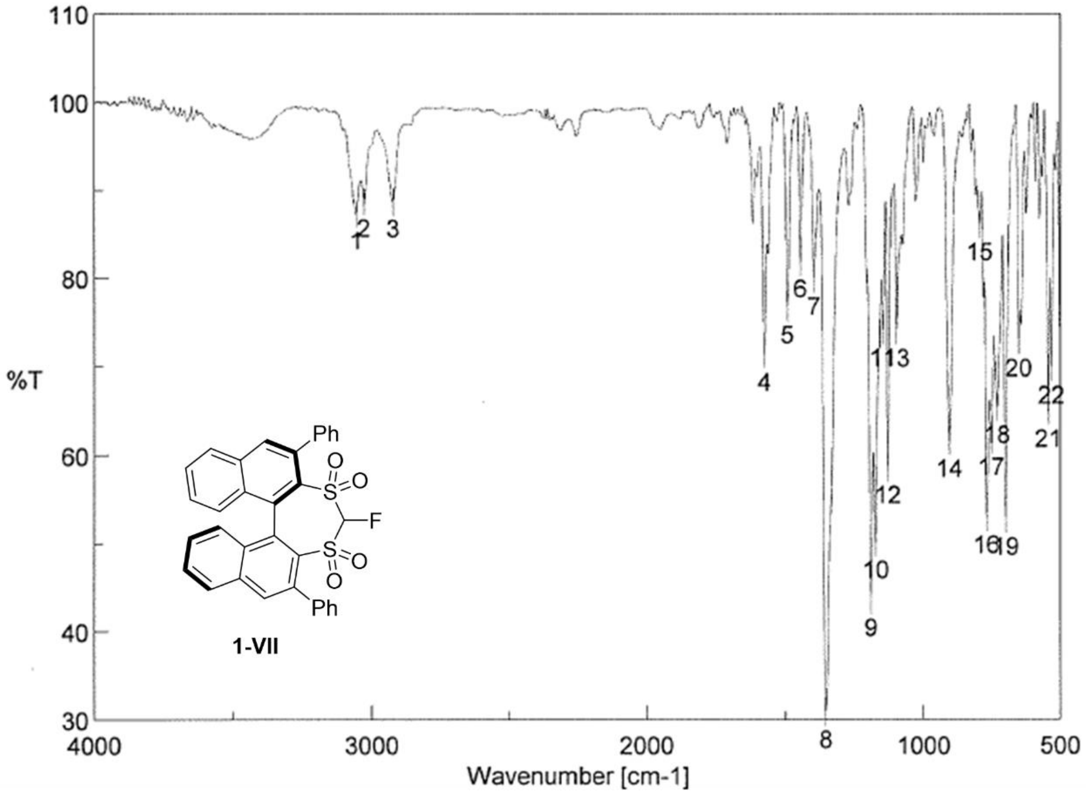

[information]
sample : 1-VII
comment : KBr

[observed peaks]

No.	Position	Strength	No.	Position	Strength
1	3055.66	87.296	2	3028.66	88.719
3	2923.56	88.691	4	1574.59	71.340
5	1492.63	76.650	6	1445.39	81.874
7	1396.21	79.874	8	1353.78	30.846
9	1189.86	43.497	10	1173.47	50.112
11	1146.47	74.046	12	1128.15	58.617
13	1097.3	74.023	14	905.415	61.622
15	795.493	86.075	16	769.458	52.952
17	752.102	61.775	18	730.889	65.412
19	699.069	52.712	20	650.858	72.885
21	541.899	64.985	22	531.293	69.847

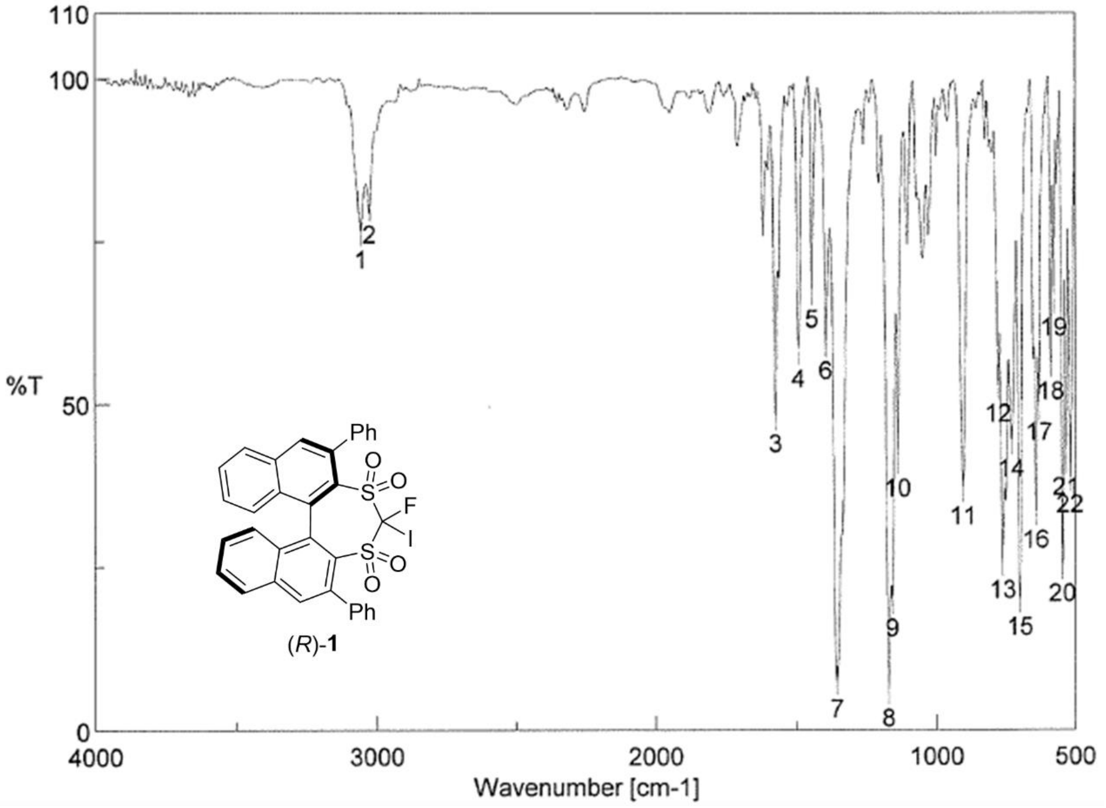

[information]

sample : (*R*)-1

comment : KBr

[observed peaks]

No.	Position	Strength	No.	Position	Strength
1	3055.66	76.431	2	3024.8	80.276
3	1573.63	48.193	4	1492.63	58.126
5	1444.42	67.247	6	1395.25	59.354
7	1356.68	7.512	8	1170.58	6.085
9	1158.04	19.781	10	1138.76	41.290
11	904.451	37.001	12	778.136	52.770
13	762.709	25.621	14	730.889	44.287
15	700.034	19.929	16	642.179	33.294
17	630.609	49.917	18	588.182	56.210
19	577.576	65.940	20	545.756	25.071
21	532.257	41.467	22	517.793	38.754
